# The Transcription Factors COUP-TFI and COUP-TFII have Distinct Roles in Arealisation and GABAergic Interneuron Specification in the Early Human Fetal Telencephalon

**DOI:** 10.1093/cercor/bhx185

**Published:** 2017-08-09

**Authors:** Ayman Alzu'bi, Susan J Lindsay, Lauren F Harkin, Jack McIntyre, Steven N Lisgo, Gavin J Clowry

**Affiliations:** 1 Institute of Neuroscience, Newcastle University, Newcastle upon Tyne NE2 4HH, UK; 2 Institute of Genetic Medicine, Newcastle University, Newcastle upon Tyne NE1 3BZ, UK; 3 Present address: School of Healthcare Science, Manchester Metropolitan University, UK

**Keywords:** cerebral cortex development, ganglionic eminences, hippocampus development, interneuron migration, SP8, ventral pallium

## Abstract

In human telencephalon at 8–12 postconceptional weeks, ribonucleic acid quantitative sequencing and immunohistochemistry revealed cortical chicken ovalbumin upstream promotor-transcription factor 1 (COUP-TFI) expression in a high ventro-posterior to low anterior gradient except for raised immunoreactivity in the anterior ventral pallium. Unlike in mouse, COUP-TFI and SP8 were extensively co-expressed in dorsal sensory neocortex and dorsal hippocampus whereas COUPTFI/COUPTFII co-expression defined ventral temporal cortex and ventral hippocampus. In the ganglionic eminences (GEs) COUP-TFI immunoreactivity demarcated the proliferative zones of caudal GE (CGE), dorsal medial GE (MGE), MGE/lateral GE (LGE) boundary, and ventral LGE whereas COUP-TFII was limited to ventral CGE and the MGE/LGE boundary. Co-labeling with gamma amino butyric acidergic interneuron markers revealed that COUP-TFI was expressed in subpopulations of either MGE-derived (SOX6+) or CGE-derived (calretinin+/SP8+) interneurons. COUP-TFII was mainly confined to CGE-derived interneurons. Twice as many GAD67+ cortical cells co-labeled for COUP-TFI than for COUP-TFII. A fifth of COUP-TFI cells also co-expressed COUP-TFII, and cells expressing either transcription factor followed posterior or anterio-lateral pathways into the cortex, therefore, a segregation of migration pathways according to COUP-TF expression as proposed in mouse was not observed. In cultures differentiated from isolated human cortical progenitors, many cells expressed either COUP-TF and 30% also co-expressed GABA, however no cells expressed NKX2.1. This suggests interneurons could be generated intracortically from progenitors expressing either COUP-TF.

## Introduction

Chicken ovalbumin upstream promotor-transcription factor 1 (COUP-TFI) and COUP-TFII are transcription factors that show very high degrees of homology except in the n-terminal region which includes an activation function domain (Wang et al. 1989; Ladias and Karathanasis 1991; Li et al. 2003). This suggests they may control transcription in a similar way but respond to different sets of activators or repressors. Accordingly, in developing mouse forebrain, COUP-TFs are expressed in overlapping but distinct domains; COUP-TFI in a low rostral to high caudal gradient across the whole cerebral cortex from embryonic day (E) 9.5 whereas high COUP-TFII expression is more restricted, either nested within the COUP-TFI expressing region but limited to the caudal-most neocortex, or in the caudo-medial wall where COUP-TFI expression is low (Qiu et al. 1994; Takiguchi-Hayashi et al. 2004; Tripodi et al. 2004; Flore et al. 2017). Expression of both COUP-TFs is considered characteristic of the caudal ganglionic eminence (CGE) (Tripodi et al. 2004) but more precisely COUP-TFI is expressed throughout the ganglionic eminences (GEs) from E10.5 to E12.5 before becoming restricted to the corticostriatal boundary, dorsal medial GE (MGE) and ventral CGE, by E13.5 (Lodato et al. 2011).

In mouse, the high expression of COUP-TFI caudally plays an important role in cortical arealisation in both progenitor and postmitotic cells by suppressing frontal cortex associated gene expression (Armentano et al. 2007; Faedo et al. 2008; Borello et al. 2014; Alfano et al. 2014a) and has been shown to have a number of roles in defining the functional identity of cortical neurons. For instance, it suppresses the differentiation of corticospinal motor neurons in the caudal somatosensory cortex, allowing for their correct specification in frontal cortex (Tomassy et al. 2010). COUP-TFI promotes faster radial migration of newborn glutamatergic neurons toward the cortical plate (CP) (Alfano et al. 2011) ensuring that concomitant axon outgrowth from these caudal/temporal cortical neurons happens in time to meet developmental deadlines such as when callosal axons are permitted to cross the midline. The present study set out to confirm that *COUP-TFI* mRNA is expressed in a posterior high/anterior low gradient in human as in rodents (Ip et al. 2010) and investigate its expression at the cellular level and in comparison with COUP-TFII.

Similarly, COUP-TFI and COUPT-TFII showed distinct expression patterns in mouse GEs suggesting a role in specifying gamma amino butyric acidergic (GABAergic) forebrain neuron phenotype. Conditional loss of COUP-TFI function in the intermediate progenitor and postmitotic interneurons alters the balance between CGE- and MGE-derived cortical interneurons without reducing their total number. CGE-derived interneurons were significantly decreased, whereas the number of MGE-derived expressing interneurons increased (Lodato et al. 2011). It is proposed that before E13.5, COUP-TFI expression in the MGE inhibits division of progenitor cells, and at later stages promotes production of CGE-derived interneurons. COUP-TFII expression is largely restricted to CGE-derived interneurons (Kanatani et al. 2008; Miyoshi et al. 2010).

Both transcription factors control rate and direction of cell migration (Tripodi et al. 2004) by regulating expression of molecules crucially involved in cell migration such as neuropilins (Tang et al. 2012) and chemokines and their receptors (Boudot et al. 2014) important in controlling tangential migration of interneurons and Cajal-Retzius cells (Stumm et al. 2003; Borrell and Marín 2006). COUP-TFI and COUP-TFII are expressed in different populations of cells in the GE; COUP-TFI in cells following dorsal (to cortex) and ventro-caudal (to diencephalon) migratory pathways (Tripodi et al. 2004) and COUP-TFII in caudally migrating cells from the CGE to the most posterior part of the telencephalon (Yozu et al. 2005; Kanatani et al. 2008; Faux et al. 2012), however, conditional knockdown of COUP-TFII has no effect upon caudal migration of cortical interneurons, which appear to maintain expression of COUP-TFI instead (Tang et al. 2012). Therefore, the present study aimed to explore the extent to which expression of the COUP-TFs in the human GE mirrors that of the rodent models, which migratory pathways cells expressing these and related markers follow, and the extent to which cells co-express COUP-TFI and COUP-TFII.

Finally, we have already demonstrated that there exists a COUP-TFII+ progenitor zone in the ventro-temporal cortex and ventro-medial frontal cortex in human (Alzu'bi et al. 2017). Whether or not this could give rise to GABAergic interneurons as well as glutamatergic pyramidal neurons remained an open question. Therefore, we isolated and cultured progenitor cells from anterior and posterior cortex avoiding temporal cortex where it adjoins the ventral CGE. We then differentiated neurons from these progenitors to see if we produced cells that expressed GABA, COUP-TFs or both.

## Materials and Methods

### Human Tissue

Human fetal tissue from terminated pregnancies was obtained from the joint MRC/Wellcome Trust-funded Human Developmental Biology Resource (HDBR, http://www.hdbr.org; Gerrelli et al. 2015). All tissue was collected with appropriate maternal consent and approval from the Newcastle and North Tyneside NHS Health Authority Joint Ethics Committee. Fetal samples ranging in age from 8 to 12 PCW were used. Ages were estimated from foot and heel to knee length measurements according to Hern (1984).

**Figure 1.**
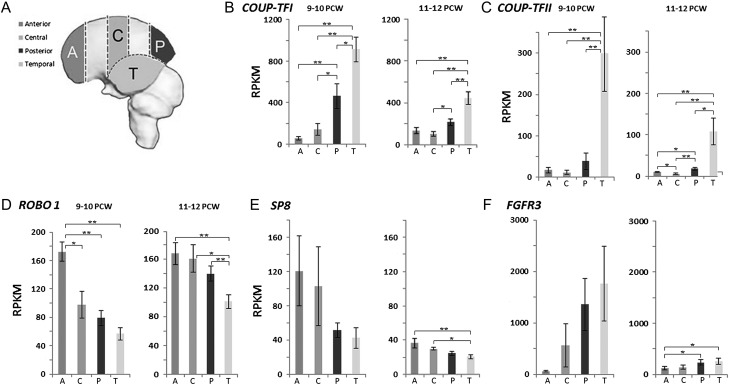
Gradients of *COUP-TFI*, *COUP-TFII*, *ROBO1*, *SP8*, and *FGFR3* expression across the cortex by RNAseq. (*A*) The location of four cortical sampling cortical regions; anterior (A), central (C), posterior (P), and temporal (T). (*B*) *COUP-TFI* expression significantly higher in temporal and posterior regions at all ages. (*C*) Highest expression of *COUP-TFII* in the temporal regions, with no significant difference in the expression between anterior, central, and posterior regions at 9–10 PCW, although there were differences at 11–12 PCW. (*D* and *E*) *ROBO1* and *SP8* both expressed in decreasing anterior posterior gradients. (*F*) *FGFR3* expressed in increasing anterior posterior gradient. Asterisk(s) represent statistically significant differences between regions (1-way ANOVA, Tukey's post hoc comparison, *P < 0.05, **P < 0.01). Error bars represent the standard error of the mean.

For ribonucleic acid quantitative sequencing (RNAseq), whole fetal brains were isolated from the skull and the meninges were removed. The hemispheres were separated and the choroid plexus and subcortical structures removed. One or both hemispheres was then divided into 6 sections. Each hemisphere represented an independent sample. The temporal lobe, including lateral and medial walls was removed and labeled Section 6. The remaining cortex was divided into 5 sections of equal width from the anterior (A) to the posterior (P) pole of the cortex including lateral and medial cortical walls (labeled 1–5). Sections 1, 3, 5, and 6 were used for RNA extraction and corresponded to anterior, central (C), posterior and temporal (T) regions (see Fig. 1, Supplementary Table 1).

For immunostaining, brains were isolated and fixed for at least 24 h at 4 °C in 4% paraformaldehyde (Sigma-Aldrich) dissolved in 0.1 M phosphate-buffered saline (PBS). Once fixed, whole or half brains (divided sagittally) were dehydrated in a series of graded ethanols before embedding in paraffin. Brain samples were cut at 8 μm section thickness in 3 different planes; horizontally, sagittally, and coronally, and mounted on slides.

### RNASeq

Full details of the origins, collection, preparation, sequencing, and analysis of the human fetal RNA samples has been previously described (Lindsay et al. 2016; Harkin et al. 2017). The entire RNAseq data set from which data was extracted for this study has been deposited at www.ebi.ac.uk/arrayexpress/experiments/E-MTAB-4840. High quality reads were then mapped to the human reference genome hg38 with Tophat2 (Kim et al. 2013). Reads aligned to genes and exons were counted with htseq-count (Anders et al. 2014) and normalized RPKMs calculated. Read length was 101 bp prior to trimming and 85 bp after trimming with no reads of less than 20 bp retained. The minimum number of reads examined per sample was 63 million (average 90 million). Mean RPKMs with standard errors were calculated for each cortical region at two developmental time points (9–10 PCW and 11–12 PCW) and compared by 1-way Anova with Tukey's post hoc test.

### Immunohistochemistry

This was carried out on paraffin sections according to previously described protocols (Alzu'bi et al. 2017; Harkin et al. 2016). Antigen retrieval involved boiling in 10 mM citrate buffer pH6 for 10 min. Sections were incubated with primary antibody (diluted in 10% normal blocking serum in Tris buffered saline [TBS] pH 7.6) overnight at 4 °C. Details of primary antibodies are found in Supplementary Table 2. Sections were incubated with biotinylated secondary antibody for 30 min at room temperature (Vector Laboratories Ltd.) 1:500 dilution in 10% normal serum in TBS followed by incubation with avidin-peroxidase for 30 min (ABC-HRP, Vector Labs) then developed with diaminobenzidine solution (Vector Labs) washed, dehydrated, and mounted using DPX (Sigma-Aldrich). For double immunofluorescence, the Tyramide Signal Amplification (TSA) method was used permitting double staining using same species antibodies. At the secondary antibody stage, sections were incubated with HRP-conjugated secondary antibody for 30 min (ImmPRESS™ HRP IgG [Peroxidase] Polymer Detection Kit, Vector Labs) and then incubated in the dark for 10 minutes with fluorescein tyramide diluted at 1/500 (TSA™) fluorescein plus system reagent (Perkin Elmer) leaving fluorescent tags covalently bound to the section. Sections were then boiled in 10 mM citrate buffer pH6 to remove all antibodies and unbound fluorescein then incubated first in 10% normal serum then with the second primary antibody for 2 h at room temperature. Sections were again incubated with HRP-conjugated secondary antibody followed by CY3 tyramide for 10 min ([TSA™] CY3 plus system reagent, Perkin Elmer). Sections were dyed with 4′,6-diamidino-2-phenylindole dihydrochloride (DAPI; Thermo Fisher Scientific) and mounted using Vectashield Hardset Mounting Medium (Vector Labs). Extensive washing of sections was carried out between all incubations.

### Dissociated Cell Culture and Immunocytochemistry

Neural progenitor cells were isolated and expanded using the neurosphere culturing method (Reynolds and Weiss 1992; Azari et al. 2011; Siebzehnrubl et al. 2011). The majority of differentiating and differentiated cells die in 2–3 days, while progenitors continue to proliferate to form neurospheres in the presence of epidermal growth factor (EGF) and basic fibroblastic growth factor (bFGF; Reynolds and Weiss 1992; Siebzehnrubl et al. 2011). Human fetal brains (*n* = 3, 9–10 PCW) were dissected under sterile conditions in Dulbecco's PBS (DPBS). The GE, anterior cortex, and posterior cortex were isolated and minced into tiny pieces before incubation in 0.05% trypsin-EDTA (Thermo Fisher Scientific) for 30 min in a 37 °C in water bath for chemical dissociation. Trypsin activity was terminated by adding the same amount of soybean trypsin inhibitor (Sigma-Aldrich). Tissue was pelleted at 110 g for 5 min, re-suspended in basal media (NeuroCult NSC Basal Medium, Stem Cell Technologies) and gently pipetted up and down until single cell suspension is achieved. The suspension was then filtered through a 70-μm-pore cell strainer (Becton Dickinson). Cells were plated at 2 × 10^5^ cells/cm^2^ into T-25 cm^2^ flasks in serum-free proliferation media (NeuroCult™ NS-A Proliferation supplements-Human, Stem Cell Technologies) and supplemented with 20 ng/mL recombinant human (rh) EGF (Sigma-Aldrich), 10 ng/mL rh bFGF (Sigma-Aldrich), and 2 μg/mL heparin (Stem Cell Technologies) for 10–15 days at 37 °C with 5% CO_2_ until the neurospheres reach 100–150 μm in diameter. Neurospheres were mechanically dissociated into single cells for passaging and/or differentiation. For cell differentiation, cells were plated at 2 × 10^5^ cells/cm^2^ onto poly-l-lysine coated glass coverslips in differentiation media (NeuroCult™ NS-A Differentiation supplements-Human, Stem Cell Technologies) for 8 days at 37 °C with 5% CO_2_. Cells were then fixed with 4% paraformaldehyde (Sigma-Aldrich) in PBS for 20 min and subjected to immunocytochemistry.

After fixation, cells were washed and blocked with 10% fetal calf serum in 0.3% Triton X-100/PBS for 1 hour at room temperature. Primary antibodies (see Supplementary Table 2) were diluted in 10% fetal calf serum in 0.3% Triton X-100/PBS and applied overnight at 4 °C. On the second day, cells were incubated with Texas Red conjugated anti-rabbit and fluorescein conjugated anti-mouse secondary antibodies (Vector Laboratories) at 1:200 dilution in PBS for 2 h at room temperature. Cells were counterstained and mounted with anti-fade mounting medium containing DAPI (Vector laboratories).

### Imaging and Cell Quantification

Images from immunoperoxidase stained sections were captured using a Leica slide scanner and Zeiss Axioplan 2 microscope; from immunofluorescent stained cells and sections with a Zeiss Axioimager Z2 apotome. Images were adjusted for brightness and sharpness using Adobe Photoshop CS6 software. Cells were counted from 5 sections from each fetal sample (8 PCW, *n* = 2 and 12 PCW, *n* = 2, therefore *n* = 10 for each age). Sections were observed under medium magnification, rectangular counting boxes of 100 μm width were placed over the ventricular/subventricular zones (VZ/SVZ) and intermediate zone/CP (IZ/CP) delineated by the nuclear staining (DAPI) on intact parts of the anterior and posterior cortex but otherwise without examining the section first. For quantification in dissociated culture, cells were cultured from 3 different fetal brains in 12-well plates, each staining combination made in duplicate. Photomicrographs were captured from 3 random fields of view from each well from which cell counts were made (therefore *n* = 18). Mean values with the standard error were calculated. Experimental groups were compared using a 2-tailed *t*-test.

## Results

### Gradients of *COUP-TFI* and *COUP-TFII* mRNA Expression Across the Developing Cerebral Cortex

Thirty-four samples of RNA were taken at 9–10 PCW and 67 at 11–12 PCW from 4 different cortical regions (Fig. 1*A*) and subjected to quantitative RNAseq analysis. Expression of *COUP-TFI* and *COUP-TFII* was compared to *FGFR3*, *ROBO1*, and *SP8*, genes predicted to show gradients of expression from previous animal experiments and human studies (Sahara et al. 2007; Zembrzycki et al. 2007; Iwata and Hevner 2009; Ip et al. 2010, 2011; Miller et al. 2014). Both *COUP-TFI* and *COUP-TFII* were expressed between 9 and 12 PCW (Fig. 1*B*, *C*), however, *COUP-TFI* was more highly expressed than *COUP-TFII* in all cortical regions and in both age groups. Significantly greater *COUP-TFI* expression was observed in the temporal/posterior compared to central/anterior cortex at 9–10 PCW (*P* < 0.05; Fig 1*B*). Although *COUP-TFI* expression decreased in the temporal and posterior cortex at 11–12 PCW, the levels still remained significantly higher in these regions (Fig. 1*B*). *COUP-TFII* showed a consistent but more confined expression pattern with significantly higher expression in the temporal lobe only. No significant difference was observed between anterior, central, and posterior regions at 9–10 PCW, although there were differences at 11–12 PCW. *COUP-TFII* expression also decreased in the temporal regions with age but remained significantly higher than in the rest of the cortex (Fig. 1*C*).

Analysis of the other 3 genes confirmed that graded expression can occur in the human cortex in both directions at these developmental stages and that the gradient seen for *COUP-TFI* was not an artifact of the experimental procedure. Both *ROBO1* and *SP8* showed expected decreasing anterior to posterior gradients (Figs. 1*D*, *E*; Ip et al. 2010; Ip et al. 2011) whereas *FGFR3* showed a distinct counter gradient similar to *COUP-TFI* (Fig. 1*F*; Ip et al. 2010; Miller et al. 2014).

### COUP-TFI Protein Expression Across the Cortex and in Relation to COUP-TFII and SP8

**Figure 2.**
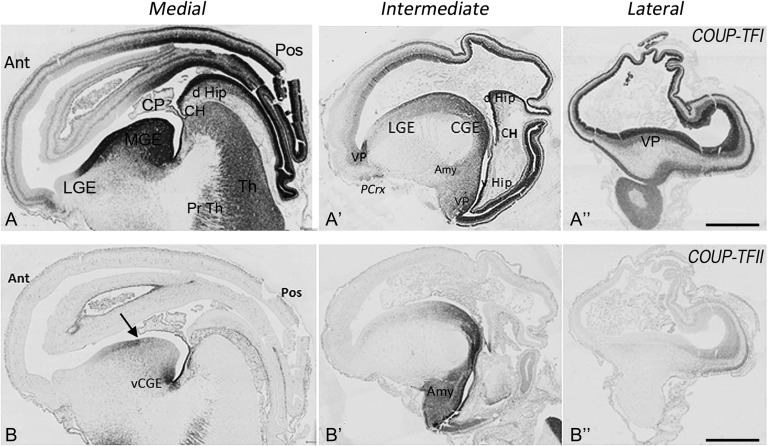
Gradients of COUP-TFI and COUP-TFII immunoreactivity across the telencephalon. (*A*). (*A*–*A*”) Parasagittal sections from medial to lateral parts of the 8 PCW fetal brain; COUP-TFI highly expressed across the GE except the dLGE, strong expression also observed in proliferative zones and CP of posterior, lateral and temporal cortex, and anterior ventral pallium. (*B*–*B*”) COUPT-FII mainly expressed in the vCGE and at the MGE/LGE boundary (arrow, B) and temporal ventral pallium (VP, B’, B”) and amygdala (Amy, B’) with dispersed scattered cells throughout rest of GE. Ant, anterior (=rostral); pos, posterior (=caudal); cent, central; Crx, cortex; PCrx, piriform cortex; CP, choroid plexus; d and vHip, dorsal and ventral hippocampus; CH, cortical hem; vCGE, ventral caudal ganglionic eminence; VP, ventral pallium; Th, thalamus; Pr Th, pre thalamus; Amy, amygdala. Scale bars = 2 mm.

**Figure 3.**
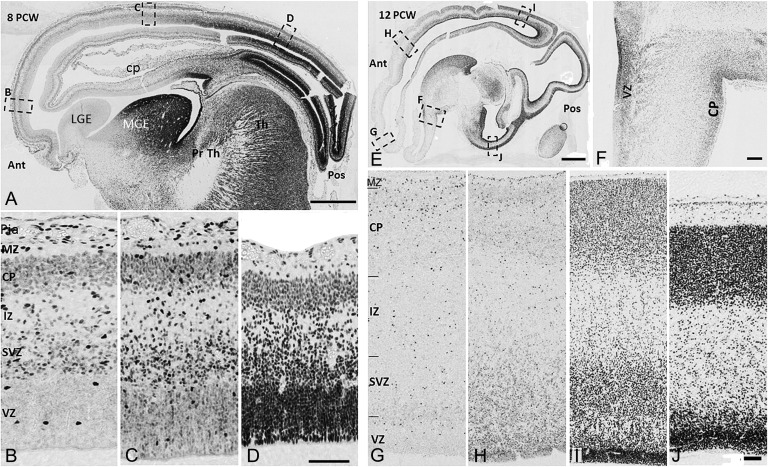
COUP-TFI immunoreactivity in the cortical wall. (*A*) Distinct posterior to anterior gradient in COUP-TFI expression across cortex in sagittal section 8 PCW. (*B*) Only a few scattered COUP-TFI+ cells observed in anterior cortex VZ, moderate to weak immunoreactivity in the SVZ/IZ/CP. (*C*) Moderate immunoreactivity across cortical wall of central cortex. (*D*) Strongest immunoreactivity in posterior cortex, relatively stronger in proliferative than in postmitotic zones. (*E*) Similar expression gradient at 12 PCW. (*F*) Strong COUP-TFI expression in proliferative and postmitotic zones of ventral pallium (VP). (*G*) COUP-TFI+ cells broadly distributed to all cortical layers of anterior cortex at 12 PCW. (*H*–*J*) COUP-TFI expression increased gradually in central, posterior, and temporal (ventral) regions, respectively. Boxed areas in A and E show where images (*B*–*D* and *F*–*J*) were taken. Ant, anterior; pos, posterior; VZ, ventricular zone; SVZ, subventricular zone; MZ, marginal zone; cp, choroid plexus; Pia, pia matter; VP, ventral pallium; Th, thalamus; Pr Th, pre thalamus. Scale bars = 500 μm in *A*, *E*; 100 μm in *D* (and for *B*, *C*); 100 μm in *F*; 100 μm in *J* (and for *G*, *H*, *I*).

**Figure 4.**
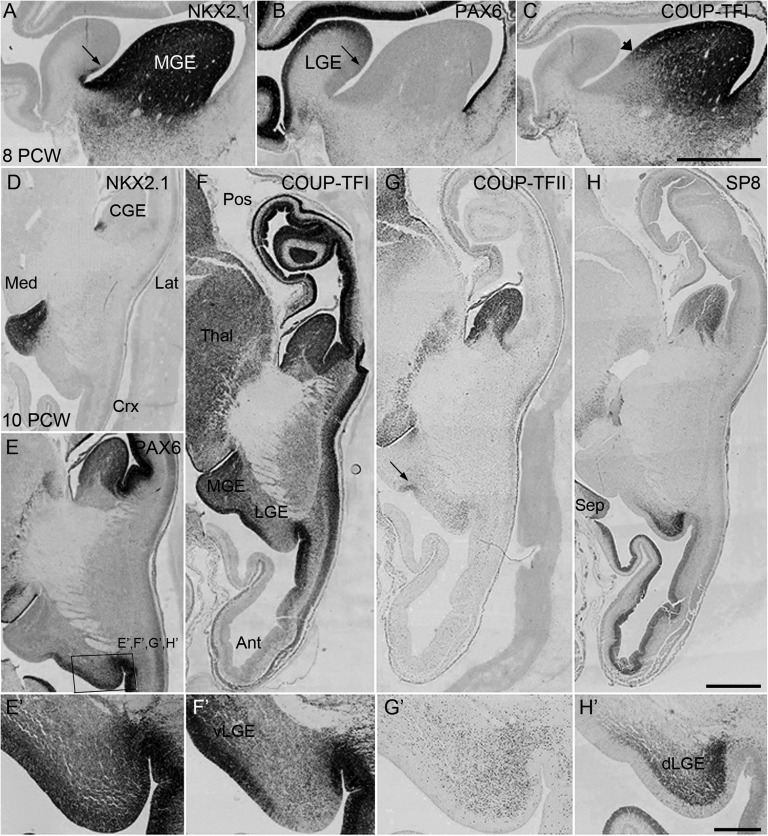
Multiple progenitor domains in the GE of early human fetal brain. (*A*–*C*) Medial parasagittal sections of 8 PCW human fetal brain. NKX2.1 expression mainly confined to MGE (*A*). PAX6 expressed in gradient with higher expression in cortical proliferative zone to lower expression in the LGE (arrows in *A* and *B* indicate boundary between MGE and LGE). (*B*) COUP-TFI showed partially overlapped expression with NKX2.1 and OLIG2 in MGE, arrow head marks the boundary between dorsal and ventral MGE (*C*). (*D*–*H*) Horizontal sections of 10 PCW human fetal brain; NKX2.1 expression confined to MGE and its caudal extension (*D*). PAX6 complementarily expressed with NKX2.1 in GE (*E*, *E*’). COUP-TFI expressed in the VZ/SVZ of the MGE, CGE, vLGE, but only in migrating cells in dLGE (*F*, *F*’). COUPT-FII highly expressed in CGE, the MGE/LGE boundary (arrowhead), and in a stream of cells entering the cortex from dLGE (*G*, *G*’). SP8 expressed in the SVZ of CGE, and in an increasing gradient from vLGE to dLGE (*H*, *H*’). Boxed areas in *E* shows where images (*E*’–*H*’) were taken. Ant, anterior; pos, posterior; Lat, lateral, Med, medial; Crx, cortex; IC, internal capsule; Sep, septum; v and d LGE, ventral and dorsal LGE; Th, thalamus. Scale bars = 500 μm in *A*–*H*, 200 μm in *E*’–*H*’.

In sagittal sections from across the medio-lateral axis of 8 PCW human telencephalon, COUP-TFI immunoreactivity was seen to increase along an anterio-medial to posterio-lateral gradient (Figs 2*A*-*A*”, 3*A*) consistent with the previous reports in rodents (Armentano et al. 2007; Borello et al. 2014). This gradient was not confined to the proliferative ventricular (VZ) and subventricular (SVZ) zones of the cortical wall, but was also observed in the postmitotic IZ and CP (Figs. 3*A*–*D*). Cellular COUP-TFI immuno-labeling was found in all layers of the posterior cortical wall, however, relatively stronger expression was observed in proliferative compared with the postmitotic zones. A gradual decrease in COUP-TFI immunoreactivity was seen through the central to the anterior cortex (Figs 3*B*–*D*). COUP-TFI expression in the VZ of the anterior cortex was restricted to only a few scattered cells; however, weak to moderate immunoreactivity was observed in many cells of the SVZ/IZ/CP (Fig. 3*B*). Interestingly, in the ventral pallium (VP; developing cortex immediately adjacent to the lateral GE (LGE)) there was strong expression of COUP-TFI but not COUP-TFII in both the proliferative and postmitotic zones even in more anterior regions (Fig. 2*A*’, 2*B*’). COUP-TFI and COUP-TFII were also expressed in the cortical hem, choroid plexus, and pia matter but with no apparent gradients across these structures (Figs 2*A*-*A*”, 2*B*-*B*’, 3*A*). A COUP-TFI gradient was maintained at later stages (10–12 PCW) along with strong expression in the VP (Figs 3*E*–*J* and 4*G*). However, in the cortical wall of the anterior cortex scattered COUP-TFI+ cells were localized to all layers by 12 PCW including occasionally in the VZ (Fig. 3*G*). In general, COUP-TFI protein expression consistently reflected the mRNA expression level seen by RNAseq analysis. Furthermore, coronal sections revealed a pronounced decreasing ventral to dorsal gradient in both the proliferative and postmitotic zones (Fig. 3*I*, *J*).

COUP-TFI positive (+) cells showed a distinct patterns of co-localization with 3 cortical cell type-specific markers paired box 6 (PAX6) (radial glia) TBR2 (intermediate progenitors) and T-box brain 1 (TBR1) (postmitotic pyramidal neurons; Bayatti, Moss et al. 2008) in different cortical areas (Supplementary Fig. 1). In posterior cortex, most COUP-TFI+ cells double-labeled with PAX6, TBR2 or TBR1, however, a small proportion expressed COUP-TFI alone (Supplementary Figs 1*A*’–*C*’). In contrast, the majority of COUP-TFI+ cells in the anterior cortex did not co-localize with cortical markers (Supplementary Figs 1*A*–*C*). Double immunofluorescence with the cell division marker KI67 (Scholzen and Gerdes 2000) revealed that most actively proliferating COUP-TFI+ cells were located posteriorly, however a few were seen in the anterior cortex (Supplementary Figs 2*B*, *C*). Thus, in posterior/temporal cortex COUP-TFI is expressed by both progenitor cells and postmitotic pyramidal neurons. In the anterior cortex, COUP-TFI is almost entirely confined to a few postmitotic cells likely to be of subcortical origin.

**Figure 5.**
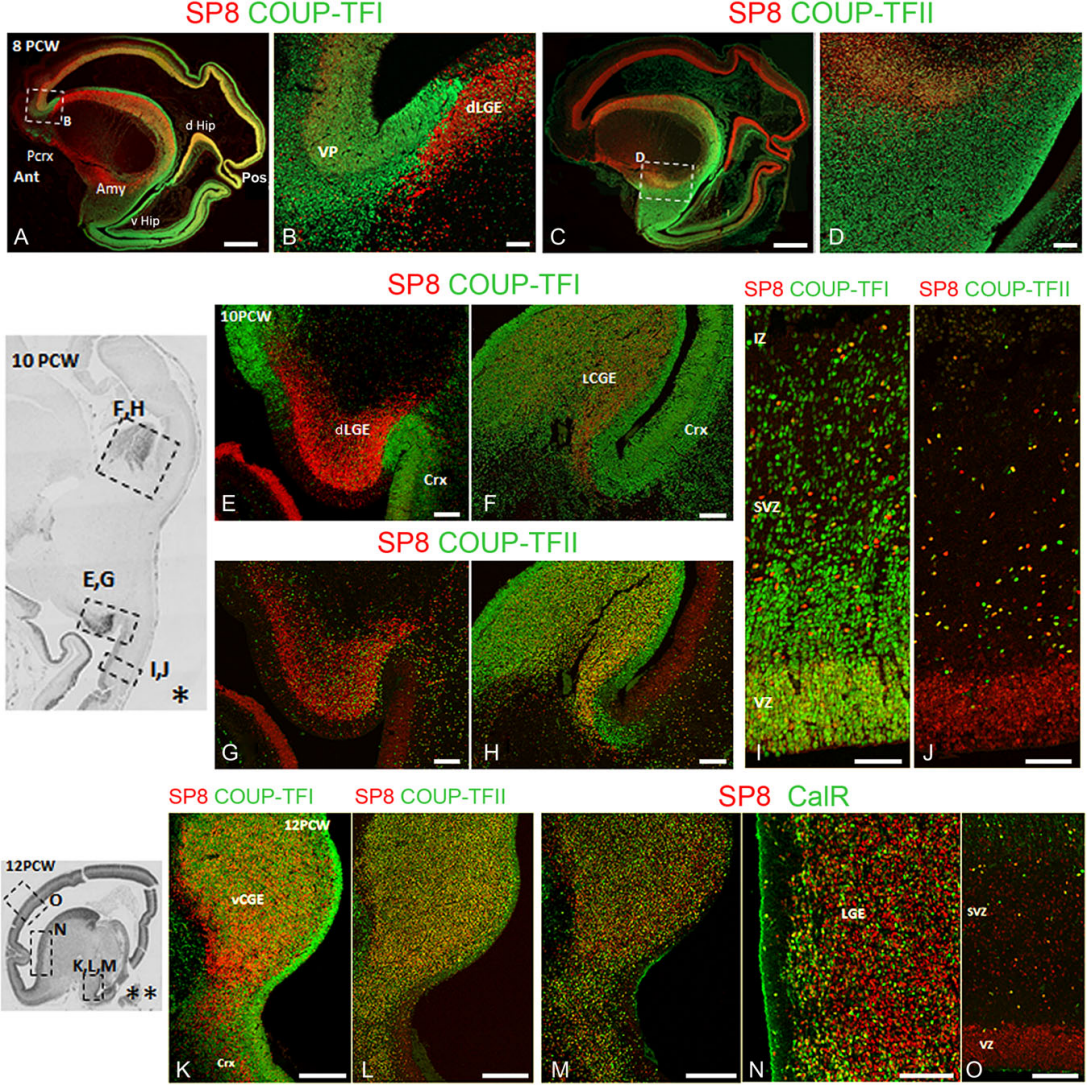
(*A*) Counter gradients of SP8 and COUP-TFI expression in cortical VZ broadly overlapped (yellow) including in posterior cortex (Pos) and the hippocampal primordium dorsal to the cortical hem (dHip). Only anterior cortex (Ant) predominantly expressed SP8, and only ventral temporal cortex including ventral hippocampus (vHip) exclusively expressed COUP-TFI. Likewise counter gradient of SP8 expression in VZ and COUP-TFI in SVZ was apparent in GE. The medial amygdala (Amy) predominantly expressed SP8, whereas ventral pallium (VP) and piriform cortex (PCrx) expressed COUP-TFI. (*B*) COUP-TFI expression in the anterior ventral pallium (VP) with SP8 confined to LGE. (*C*) In cortex SP8 and COUP-TFII show abrupt expression boundaries; COUP-TFII confined to ventral temporal lobe. (*D*) Posterior regions of medial amygdala populated by SP8+/COUP-TFII+ cells. (*E*) the dLGE/cortex boundary was sharply delineated by SP8 and COUP-TFI expression. (*F*) Relatively small numbers of SP8+/COUPTFI- cells migrate from LGE-Like CGE (LCGE) into the cortex at this stage. (G) Low density of COUP-TFII+ cells in SP8+ dLGE. (H) High density of SP8 + /COUP-TFII+ cells in SVZ of the LGE-Like CGE. (I and J) SP8+/COUP-TFI+ cells predominantly seen in cortical VZ although double-labeled cells present in SVZ and IZ (*I*). SP8+/COUP-TFII+ double-labeled cells predominantly observed in SVZ and IZ (*J*). (*K* and *L*) SP8 Co-expressed with both COUP-TFI and COUP-TFII in ventral CGE. (*M*, *N*, *O*) Extensive co-expression of SP8 and CalR in the vCGE (M) but also in the LGE and cortex (*N*, *O*). Crx, cortex; d and vHip, dorsal and ventral hippocampus; Amy, amygdala; VP, ventral pallium; dLGE, dorsal LGE; L and vCGE, lateral and ventral CGE; VZ, ventricular zone. Scale bars = 2 mm in *A*, *C*; 500 μm in *K*, *L*, *M*; 200 μm in *E*–*H*, *N*, *O*; 100 μm in *B*, *D*, *I*, *J*.

Finally, COUP-TFII immunoreactivity expressed in the ventro-temporal region nested within the larger COUP-TFI expressing domain (Figs. 2*B*–*B*”). COUP-TFI showed a complementary expression pattern to SP8 immunoreactivity which was expressed in a counter gradient as previously described in rodents (Sahara et al. 2007; Zembrzycki et al. 2007; Fig. 5 and Supplementary Figs 5*A*, *F*). Interestingly, whereas there was extensive overlap of expression of COUP-TFI and SP8 in parietal, dorso-posterior, dorso-temporal, and dorsal hippocampus (Fig. 5*A*) SP8 and COUP-TFII expression formed sharp boundaries with SP8 excluded from the ventral posterior and temporal cortex including the ventral hippocampus (Fig. 5*C*). Unlike COUP-TFI and COUP-TFII, SP8 was downregulated in postmitotic neurons in the CP (Figs 5*A*, *C*). These observations are in accord with the RNAseq data (above) and previous observations by immunohistochemistry (Ma et al. 2013; Alzu'bi et al. 2017) but these double labeling experiments throw light on possible interactions between the 3 transcription factors in human cortical development.

### COUP-TFI and COUP-TFII Differentially Expressed in the Subdivisions of the GEs

COUP-TFI immunoreactivity was mapped to the subdivisions of the GE revealed by the expression patterns of 3 transcription factors (PAX6, NK2 homeobox 1 (NKX2.1), oligodendrocyte lineage transcription factor 2 (OLIG2); Pauly et al. 2014; Alzu'bi et al. 2017; Fig. 4). When correlated with NKX2.1 and OLIG2 expression, COUP-TFI immunoreactivity revealed 2 distinct neurogenic domains in the MGE; 1 large dorsal domain characterized by overlapped intense cellular expression of COUP-TFI, NKX2.1, and OLIG2 in the proliferative zone (dMGE) and a smaller ventral domain (vMGE) characterized by strong expression of NKX2.1 and OLIG2 only (Figs 4*A*–*D*). A proportion of COUP-TFI+ cells in the dMGE co-expressed NKX2.1 (Supplementary Fig. 3*A*,*B*) and OLIG2 (Supplementary Fig. *3C*). Intense COUP-TFI expression marked the proliferative zones at the MGE/LGE boundary and the ventral part of the LGE (vLGE; Figs 4*G*, *G*’). In the dorsal LGE (dLGE) COUP-TFI expression was limited to a few scattered cells in the postmitotic mantle at 8 PCW (Fig. 4*D*) however, more cells appeared here at 10 and 12 PCW; possibly cells migrating into the cortex (Fig. 4*G*’). In the CGE, COUP-TFI+ cells were present throughout the proliferative zones of d and vCGE (Figs 3*E*, 4*G*). Co-localization of COUP-TFI with KI67 showed that proliferating COUP-TFI+ cells were found in the dMGE, CGE, and vLGE, but not in the dLGE (Supplementary Fig. 2).

**Figure 6.**
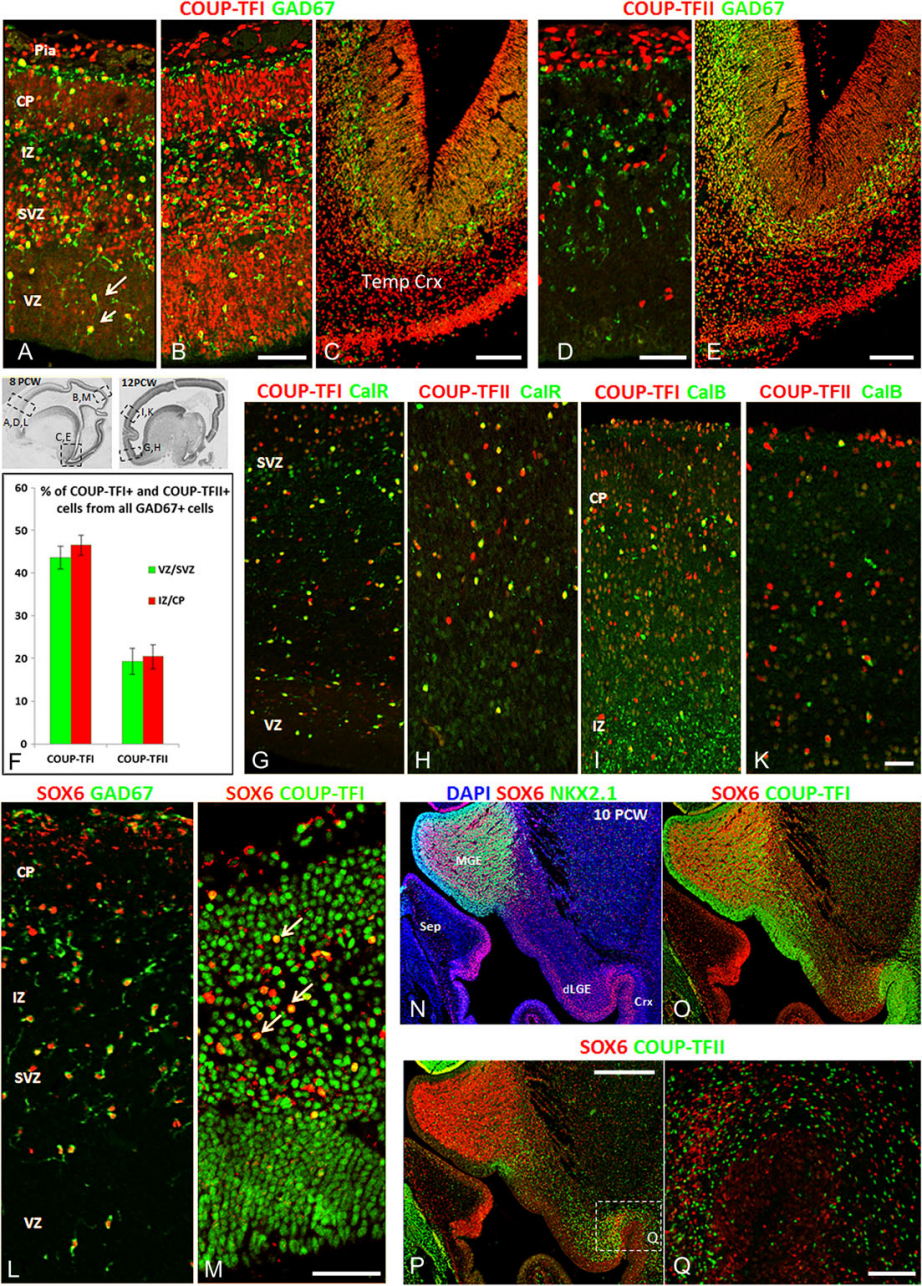
COUPT-FI and COUP-TFII expression in cortical GABAergic interneurons. (*A*–*C*) Double labeling for COUPT-FI and GAD67 in cortical wall at 8 PCW. COUP-TFI+/GAD67+ cells broadly distributed in the cortical wall, mostly either just above the thin CP in the marginal zone or just below in the pre-subplate/IZ; (*A*, *B*) A considerable number also found in proliferative (VZ/SVZ) zone of anterior and posterior cortex with radial nuclear morphology (arrows). (*C*) A stream of COUP-TFI+/GAD67+ cells enter temporal cortex from vCGE, migrating tangentially mainly in the SVZ/IZ. (*D*, *E*) A smaller proportion of GAD67+ cells co-express COUP-TFII. (*F*) The percentage of COUP-TFI+ and COUP-TFII+ cells from all GAD67+ cells in the proliferative (VZ/SVZ) and postmitotic zones (IZ/CP) of 8 PCW cortical wall. (*G*, *H*) Double labeling for COUPT-FI and COUP-TFII with calretinin (CalR) in cortical wall of 12 PCW fetal brain. (*I*, *K*) Double labeling for COUPT-FI and COUP-TFII with calbindin (CalB) in 12 PCW cortical wall. (*L*, *M*) Double labeling for SOX6 and GAD67 in the 8 PCW cortical wall. The majority of SOX6+ cells co-expressed GAD67 this stage (*L*), while a proportion of SOX6+ cells also co-expressed COUP-TFI (*M*). (*N*) Double labeling for SOX6 and NKX2.1 in the GE of of 10 PCW fetal brain. SOX6 mainly expressed in NKX2.1+ cells in SVZ of MGE and in migrating cells through the LGE mantle zone; SOX6 also expressed in the cortical and dLGE VZ. (*O*–*Q*) Double labeling for COUPT-FI or COUP-TFII with SOX6 in GE at 10 PCW; COUP-TFI highly expressed in SOX6+ cells in MGE and in cells entering cortex from LGE (*O*), no similar double labeling with COUP-TFII (*P*, *Q*). Insets with boxed areas show where images (*A*–*E* and *G*–*M*) were taken. Images *N*–*Q* are taken from a similar section to Figure 4*I*. VZ, ventricular zone; Pia, pia matter; Temp crx, temporal cortex; Sep, septum; dLGE, dorsal LGE. Scale bars = 100 μm in *B*, *D* (and for *A*); 200 μm in *C*, *E*; 100 μm in *K* (and for *G*, *H*, *I*); 100 μm in *M* (and for *L*) 500 μm in *P* (and for *N*, *O*); 100 μm in *Q*.

Double immunofluorescence histochemistry revealed co-expression of COUP-TFI and COUP-TFII by cells in both the cortex and GEs (Supplementary Fig. 4). Throughout both compartments COUP-TFI was more widely expressed than COUP-TFII, however, a large proportion of cells in the CGE were immunoreactive for both transcription factors (Supplementary Fig. 4*A*, *E*, *F*). Gradients of either single or double-labeled cells suggested 2 possible migratory pathways out from the CGE: posteriorly into the temporal cortex, and anterio-laterally through the LGE into the anterior and central cortical regions (Supplementary Fig. 4*B* and *E*–*H*; Touzot et al. 2016). At 8 PCW, the posterior pathway appeared predominant (Fig. 2*A*’, *B*’ and Supplementary Fig. 4*B*) however, the number of the cells that migrated anterio-laterally significantly increased by 10–12 PCW (Supplementary Fig. 4*G*). This suggests that pathway selection for cell migration out the CGE is controlled in a temporal manner. The present study also shows that, in addition to the CGE, COUP-TF+ cells could also originate from the MGE (COUP-TFI+ Fig. 6*O*) and the MGE/LGE boundary (COUP-TFI+, COUP-TFII+, and COUP-TFI+/COUP-TFII+ cells, Fig. 2*A*, *B*). At 8 and 12 PCW, respectively, 20 ± 3.6% and 22 ± 3% of all COUP-TFII+ cells in the cortex also expressed COUP-TFI. The highest proportion of double-labeled cells was observed in the ventro-temporal cortex (Supplementary Fig. 4*C*, *D* and *H*–*J*).

### SP8 Expressed in Distinct Populations of Postmitotic COUP-TF Expressing CGE and LGE-Derived Cells

In mouse, the transcription factor SP8 plays a role in the differentiation of LGE-derived interneurons that populate the amygdala and the olfactory bulb via the rostral migratory stream (RMS) (Waclaw et al. 2006). In the human ventral telencephalon, SP8 was expressed in the SVZ of the LGE with an increasing gradient from ventral to dorsal, in the septum and in the RMS (Fig. 4*I*, *I*’ and Supplementary Fig. 5). Two streams of SP8+ cells were observed migrating ventrally and rostrally into the RMS from both the dLGE and septum (Supplementary Fig. 5*C*, *E*) suggesting the septum also contributes interneurons to the human olfactory bulb. SP8 was also highly expressed across the SVZ of the vCGE (Supplementary Fig. 5*A*, *B*) not only in the caudal extension of the LGE (LCGE) as previously described (Ma et al. 2013). A large number of SP8+ cells formed a migratory stream from the vCGE ventrally toward the developing amygdala. Although few SP8+ cells were seen entering the cortex from the CGE at 8 PCW, the number increased at 12 PCW (Supplementary Fig. 5*B*, *G*).

In either LGE or CGE, SP8 was predominately expressed in COUP-TFII+ and to a lesser extent in COUP-TFI+ cells mostly located in the SVZ and appearing to migrate tangentially into the cortex (Fig. 5*A*–*H*, *K*–*L*). COUP-TFII+/SP8+ in cells appeared to migrate ventrally from the vCGE into the posterior part of the mantle zone lateral and ventral to the GE (Figs. 5*C*, *D*). However, SP8 was also expressed in the anterior part of this region where these cells appeared to be migrating from the LGE (Fig. 5*C*). In the cortical wall both COUP-TFI+/SP8+ and COUP-TFII+/SP8+ cells were mainly found in the SVZ and IZ; few were found in the CP (Figs. 5*I*, *G*). As SP8 was not expressed in the MGE or at the MGE/LGE boundary, cortical COUP-TFI+/SP8+ cells and COUP-TFII+/SP8+ cells were most likely generated in the CGE (not the LGE) migrating either posteriorly or anterio-laterally into the cortex, confirmed by observing that a large proportion of SP8+ cells in these 2 pathways co-expressed CalR (Figs 5*M*–*O*), a marker of CGE-derived interneurons (Miyoshi et al. 2010). However, we also found a population of SP8+ cells entering the cortex from the dLGE, similar to Ma et al. (2013) except that these cells did not co-express COUP-TFI, COUP-TFII, or CalR (Fig. 5).

### COUP-TFI Expressed by both MGE- and CGE-Derived Cortical GABAergic Interneurons

The majority of COUP-TFI+ cells in the anterior cortex expressed the GABA synthesizing enzyme glutamate decarboxylase 67Kd (GAD67). As expected, a smaller proportion of COUP-TFI+ posteriorly expressed GAD67 because a far higher density of COUP-TFI+ cells co-expressed markers for glutamatergic neurons and their precursors (TBR1, PAX6 and TBR2; see above and Figs 6*A*,*B*, Supplementary Fig. 1). In the cortex at 8 PCW GAD67+ cells were mostly found in either the IZ/SP or the MZ, the 2 major migration streams of GABAergic interneurons in the developing cortex (Marín 2013); however, a considerable number of GAD67+ cells also appeared to be migrating in the VZ/SVZ (Fig. 6*A*, *B*). COUP-TFI/GAD67 co-localization was also observed in all these compartments with 47 ± 2.7% of all GAD67+ cells in the proliferative zones (VZ/SVZ) co-expressing COUP-TFI. In particular, the majority of GAD67+ cells migrating in the VZ co-expressed COUP-TFI with some exhibiting a longitudinal morphology suggesting radial migration. A similar proportion was also seen in postmitotic layers (45 ± 2.4%) located either at the CP/MZ border or just below in the pSP/ IZ (Fig. 6*A*–*C*, *F*).

NKX2.1 is downregulated in migrating MGE-derived cells before they enter the cortex; SRY-box6 (SOX6) acts downstream of NKX2.1 and its expression is maintained in migrating interneurons in mouse (Batista-Brito et al. 2009). At 8 PCW, SOX6 was expressed in the SVZ of the MGE, in cells probably migrating through the LGE, and in the cortex. SOX6+ cells were also broadly distributed throughout the cortical wall, the majority of which co-expressed GAD67 (Fig. 6*L*) and a proportion of which also co-expressed COUP-TFI (Fig. 6*M*) confirming that a population of COUP-TFI+ cortical interneurons are derived from the dMGE. This was apparent at 10 PCW, where COUP-TFI+ cells showed co-localization with SOX6 in the SVZ of the MGE and in a large number of cells migrating into the cortex from the LGE (Fig. 6*O*). Moderate SOX6 immunoreactivity also appeared in the cortical and dLGE VZ at this stage (Fig. 6*N*) suggesting, as in rodents, a role in determining cortical progenitor identity (Azim et al. 2009).

In addition, other interneurons expressing COUP-TFI were characterized by co-expression of either calretinin (CalR) or calbindin (CalB; Figs 6*G*, *I*). CalR, in particular, is characteristic of CGE-derived cortical GABAergic interneurons in rodents (Kanatani et al. 2008). At 12 PCW, when the cortical wall was extensively populated with CalR+ cells, we found a subpopulation of these cells co-localized COUP-TFI (Fig. 6*G*). Overall 39 ± 4.7% of all CalR+ cells in all cortical regions co-expressed COUP-TFI. Although there were fewer CalB+ cells present at this stage, a proportion of these also co-expressed COUP-TFI (Fig. 6*I*).

### COUP-TFII Expressed Mainly by CGE-Derived Cortical GABAergic Interneurons

The expression of COUP-TFII by a subpopulation of cortical GABAergic interneurons was also analyzed by double immunofluorescence with GAD67, CalR, CalB, and SOX6 (Fig. 6). Either in the proliferative zone or postmitotic layers, a far smaller proportion of GAD67+ cells expressed COUP-TFII than COUP-TFI; only 19 ± 3% of all GAD67+ cells expressed COUP-TFII in the proliferative zone (mainly in the SVZ), many COUP-TFII+ cells were seen in the VZ, but these cells did not co-express GAD67. In the postmitotic layers, 20 ± 2.8% of GAD67+ cells expressed COUP-TFII and were also mostly seen in the IZ/SP and above the CP in the marginal zone (Fig. 6*D*–*F*). Conversely, a higher proportion of COUP-TFII+ cells co-localized CalR (Fig. 6*H*); 67 ± 6% of all CalR+ cells in the cortex of 12 PCW human brain co-expressed COUP-TFII, however, a proportion of COUP-TFII+/CalR+ cells are probably Cajal-Retzius cells (Zecevic et al. 2011; González-Gómez and Meyer 2014). Some COUP-TFII+ cells in the cortex were also immunoreactive for CalB (Fig. 6*K*). Although the MGE/LGE boundary could also be an origin for COUP-TFII+ cells (Fig. 2*B*; Alzu'bi et al. 2017); COUP-TFII was not co-expressed with SOX6 (Fig. 6*P*,*Q*). However, COUP-TFII+ interneurons in the cortex can co-express OLIG2 (Reinchisi et al. 2012) and these cells could be derived from the MGE/LGE boundary.

### COUP-TFI, COUP-TFII, and GABA Immunoreactive Cells in Dissociated Cultures from Human Fetal Cortex

**Figure 7.**
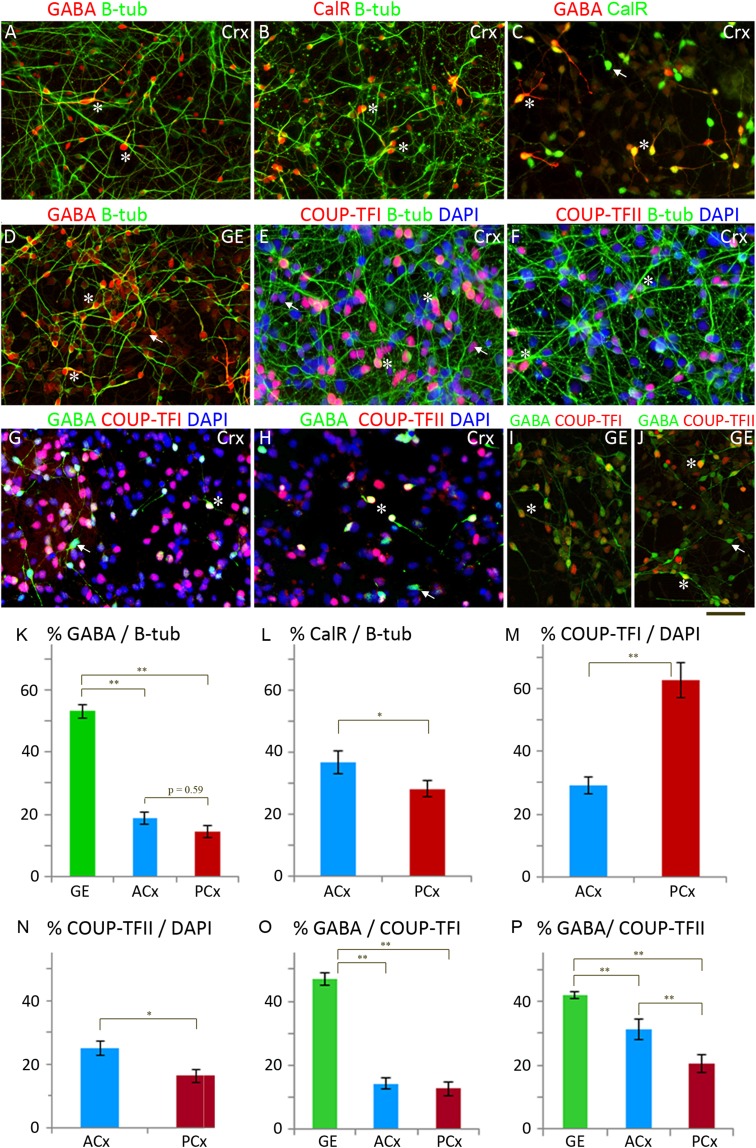
Examples of double immunofluorescent staining of cultured cells derived from human fetal cortex (Crx, *A*–*C*, *E*–*H*) and GEs (*GE*, *D, I, J*) showing that GABA+ neurons can be generated from cortically-derived progenitor cells. Counts were made from these cultures and the results plotted as bar charts; for instance K shows the% of B-tubulin expressing neurons expressing GABA in cultures derived from GE anterior cortex (ACx) posterior cortex (PCx). Error bars represent the standard error of the mean, 1 asterisk denotes a statistically significant difference *P* < 0.05, 2 asterisks *P* < 0.0005, 2-tailed *t*-test.

Cortical cell cultures, prepared by isolating and expanding cortical progenitor cells followed by a differentiation protocol, were first characterized by immunocytochemistry for markers of human cortical neural progenitor cells (KI67, PAX6; Bayatti, Moss et al. 2008) astrocytes/older radial glia (GFAP; Levitt and Rakic 1980) postmitotic neurons (b-tubulin; Katsetos et al. 2003) and cortically-derived glutamatergic neurons (TBR1; Bayatti, Moss et al. 2008). Mixed populations of progenitor and postmitotic cells were found. 21 ± 1% of the total number of DAPI labeled cells expressed the cell division marker KI67, 25 ± 2% PAX6 and 3 ± 0.02% GFAP, and 35 ± 1% expressed b-tubulin (data not shown). Of note, 67 ± 3% of B-tubulin expressing neuroblasts/neurons were also TBR1 positive (data not shown), but 17 ± 2% were immunoreactive for GABA (19 ± 2% in Ant Crx and 14 ± 2% in Pos Crx; Fig. 7*A*, *K*) and 32 ± 2% CalR (37 ± 4% in Ant Crx and 28 ± 2% in Pos Crx; *P* < 0.05; Fig. 7*B*, *L*). However, only 49 ± 3% of CalR+ cells co-expressed GABA (Fig. 7*C*) confirming that CalR expression in human fetal brain is not restricted to GABAergic interneurons but is also a marker for cortically-derived pioneer neurons and Caja-Retzius cells (González-Gómez and Meyer 2014).

As GABAergic interneurons are proposed to derive almost entirely from progenitor cells of the GEs in primate (Hansen et al. 2013; Ma et al. 2013; Arshad et al. 2016) these observations may indicate either that cells have lost their regional identity during dissociation, expansion, and culturing or that a proportion of interneurons are generated from cortical progenitor cells in developing fetal human brain as previously suggested (Zecevic et al. 2011; Radjonic, Ayoub et al. 2014; Clowry 2015). There was evidence that the cultures retained regional identity. First, cultures of GE derived cells from the same brains showed a far higher proportion of b-tubulin+/GABA+ cells (53 ± 2%, Fig. 7*D*, *K*) and a proportion of cells expressed NKX2.1, characteristic of MGE-derived progenitors, whereas no NKX2.1 immunoreactivity was observed in cortical cultures as seen in forebrain sections immunostained for NKX2.1 before 12 PCW (Hansen et al. 2013; Pauly et al. 2014; Alzu'bi et al. 2017). Second, COUP-TFI was expressed differentially between anteriorly and posteriorly derived cortical cultures (Ant Crx and Pos Crx) as it is between anterior and posterior cortex; 29 ± 3% of all cells in Ant Crx cultures and 63 ± 6% of cells in Pos Crx cultures (Fig. 7*E*, *M*). A proportion of COUP-TFI+ cells were double-labeled with either PAX6 or B-tubulin, indicating that COUP-TFI was expressed in both progenitor and postmitotic cortical cells (not shown). A lower level of expression was found for COUPTFII than for COUP-TFI (Fig. 7*F*, *N*). All these observations are consistent with the immunohistochemical and RNAseq data from intact brains (see above). Furthermore, and contrary to the findings for COUP-TFI expression, the proportion of DAPI labeled cells also immunoreactive for COUP-TFII+ was significantly higher (*P* < 0.05) in Ant Crx cultures (25 ± 2%) than in PCrx (16 ± 2%; Fig. 7*N*).

We hypothesized that COUP-TFI and COUP-TFII positive progenitors could also provide an origin for GABAergic interneurons born in the cortex in addition to the GE, as has been previously suggested, especially for COUP-TFII (Reinchisi et al. 2012; Radjonic, Ayoub et al. 2014; Alzu'bi et al. 2017). In Ant Crx cultures 14 ± 2% of COUP-TFI + and 31 ± 3% of COUP-TFII+ cells were co-labeled for GABA and in Pos Crx 13 ± 2% of COUP-TFI+ and 20 ± 2% of COUP-TFII+ cells were similarly double-labeled (Figs 7*G*, *H*, *O*, *P*). Although a higher proportion of cells co-labeled for GABA and COUP-TFs in GE cultures (Figs. 7*I*, *J*, *O*, *P*) the conclusion still is that at the time of culturing there were progenitors present in the cortex that express COUP-TFI and/or COUP-TFII and are capable of generating GABAergic interneurons.

## Discussion

COUP-TFI and COUP-TFII have been shown to be key regulators of telencephalic development in numerous experiments in rodents contributing to the protomap, controlling neurogenesis, determining phenotype and influencing rates and direction of cell migration (reviewed by Alfano et al. 2014b). It is important to determine the extent to which these roles have been retained or altered in human development particularly as mutations in COUP-TFI have been implicated in intellectual disability (Bosch et al. 2014). The present study confirms that these transcription factors are likely to have equally important and largely similar roles in human, however, the greater complexity of the human compared to the rodent brain has revealed small differences that may, be important in understanding differences between the species and neurodevelopmental disorders.

### COUP-TFI, COUP-TFII, and SP8 as Regulators of Human Cortical Arealisation

This study confirms that the observation that COUP-TFI and SP8 form counter gradients of expression across the mouse pallium (O'Leary et al. 2007; Rakic et al. 2009; Sansom and Livesey 2009; Borello et al. 2014; Alfano et al. 2014a) is also the case in the human cerebral cortex. A fundamental difference between mouse and primate, and in particular human, cerebral cortex is the substantially larger surface area with, more importantly, a more complicated pattern of functional arealisation and a considerably larger proportion devoted to higher functioning association cortex (Van Essen and Dierker 2007; Krubitzer and Seelke 2012; Buckner and Krienen 2013). One mechanism that could contribute to the increased complexity is more sublety in the combinatorial patterning of transcription factor expression to determine the human protomap. Here, we show that the expression of SP8 and COUP-TFI overlaps extensively in certain cortical regions, but expression of SP8 and COUP-TFII forms distinct boundaries dividing the cortical wall into regions that express SP8 only (the frontal lobe excluding anterior ventral pallium) COUP-TFI/COUP-TFII (ventral posterior and ventral temporal cortex, lateral and medial) and COUP-TFI/SP8 (parietal, dorsal posterior, dorso-temporal, and dorsomedial cortex). In the mouse, protomap COUP-TFI and SP8 show little overlap (Borello et al. 2014) and COUP-TFII is confined to a very small portion of the posterior cortex (Qiu et al. 1994) possibly the origin of secondary temporal cortex (Wree et al. 1983). Combinatorial expression of COUP-TFI and SP8 could maintain a common genetic identity for future primary sensory areas (visual, auditory, and somatosensory) and a partially shared identity with SP8-expressing frontal motor cortex with which these sensory areas will interconnect, along with allied association cortex, via dorsal sensorimotor pathways (Baker 2007; Hickock and Poeppel 2007; Milner and Goodale 2008). On the other hand, expansion of cortical COUP-TFII expressing territory in human fetal brain mirrors the increase in size and complexity, in human compared with mouse, of the association areas of the ventro-temporal cortex that includes the ventral stream of cognitive visual processing (Milner and Goodale 2008; Kaas 2013).

The dorsal (posterior in adult human) and ventral (anterior in adult human) hippocampus were also differentiated by combinatorial expression of SP8/COUP-TFI and COUP-TFII/COUP-TFI, respectively. The dorsal domain featured in our sections lies anterior and dorsal of the cortical hem but does not include that anterior-most portion that dissipates after 14 PCW as the corpus callosum develops (Kier et al. 1995). Dorsal and ventral hippocampus have distinct roles; dorsal carries out primarily cognitive functions whereas ventral hippocampus function is primarily related to stress, emotion, and affective states (reviewed by Fanselow and Dong 2010; Strange et al. 2014) reflected by distinct patterns of gene expression in adult and postnatal rodents (Dong et al. 2009; O'Reilly et al. 2015) and by distinct efferent and afferent connectivity, also demonstrated in primates (Friedman et al. 2002; Kondo et al. 2009; Aggleton et al. 2012) and suggested in human by neuroimaging (Bunsey and Eichenbaum 1996; Chua et al. 2007; Strange et al. 2014). In mouse, high to low expression of COUP-TFI along a septo-temporal gradient is important for the functional organization of the hippocampus (Flore et al. 2017). Here, we provide evidence that the protomap for human hippocampal specialization is laid down prior to formation of afferent and efferent connections, determined by complementary expression of SP8 and COUP-TFII rather than graded expression of COUP-TFI.

The ventral pallium, a cortical structure forming the boundary between the cortex and the lateral GE, contributes cells to the olfactory cortex, claustrum and amygdala in mouse (Puelles et al. 2000; Medina et al. 2004) is present in human (Lindsay et al. 2005). Here, we show it is characterized by COUP-TFI expression throughout with anterior and posterior regions defined by SP8 or COUP-TFII co-expression, respectively. In mouse where *Coup-TFI* is expressed at the anterior corticostriatal boundary (Lodato et al. 2011) and in both human and mouse there is expression of COUP-TFI in postmitotic cells of the nearby piriform olfactory cortex (Fig. 6*A*; Lodato et al. 2011).

Interaction of COUP-TFI, COUP-TFII, and SP8 with other transcription factor expression gradients demonstrated to exist in human fetal brain at this stage of development, for instance EMX2 and PAX6 (Bayatti, Sarma et al. 2008; Ip et al. 2010) CTIP2 (Ip et al. 2011) and OLIG2 (Alzu'bi et al. 2017) may produce a sufficiently complicated mosaic of organizing maps that “tether” the subsequent rapid expansion of the human cerebral surface during development to produce the complex but largely stereotyped interconnection of primary, secondary, and association cortex (Buckner and Krienen 2013). It has been proposed that the early developing medial temporal and V1 areas of the visual cortex in nonhuman primates act as “molecular anchors” for the subsequent development of the dorsal and ventral visual streams, respectively (Bourne and Rosa 2006; Homman-Ludiye and Bourne 2014). These areas could be defined in early cortex by differential expression of SP8 and COUPTFII, both combined with COUP-TFI expression.

### Compartmentalization of the Ventral Telencephalon

The proliferative zones of the ventral telencephalon can be divided according to transcription factor expression allowing some predictions as to how interneuron precursors derived from each compartment migrate into the telencephalon. COUP-TFI immunoreactivity subdivided the MGE with NKX2.1 and OLIG2 expressed throughout but with COUP-TFI confined to the larger dorsal region. In rodents the dMGE is the birthplace of nearly all parvalbumin-positive (PV+) and somatostatin-positive (SST+) cortical interneurons, whereas the vMGE predominantly gives rise to globus pallidus neurons (Flandin et al. 2010). We observed co-expression of COUP-TFI with SOX6, a downstream regulator of NKX2.1 (Batista-Brito et al. 2009) in the dMGE and in laterally migrating neuroblasts through the LGE and into the cortex, demonstrating co-expression of COUP-TFI and SOX6 in cortical interneurons in human for the first time. COUP-TFI may have a role in guiding migration (Boudot et al. 2014) perhaps ensuring neurons migrate dorsally toward the cortex.

The LGE is also divided into dorsal and ventral portions by COUP-TFI expression in the VZ ventrally. Dorsal LGE is characterized by stronger PAX6 expression, SP8 expression in postmitotic cells and few COUP-TFII+ cells. Between 8 and 12 PCW COUP-TFII immunoreactivity in the dLGE appeared to belong only to anteriorly migrating cells arising from the vCGE. Instead, dLGE provided predominantly SP8/CalR- cells that migrated toward the RMS, amygdala, and cortex. The MGE/LGE boundary zone is identified by COUP-TFI/COUP-TFII expression, distinct from the adjacent dMGE and vLGE (Fig. 2*A*,*B*, 4*G*,*H*; Ma et al. 2013; Alzu'bi et al. 2017); suggesting that this region could be an origin for COUP-TFII+ interneurons in addition to the vCGE (see below). In rodents, this boundary region is the source of COUP-TFII+/SOM+ cells that occupy cortical layer V (Cai et al. 2013). However, we found no co-expression of COUP-TFII with SOX6, the developmental marker for SOM+ interneurons, which suggests a difference with rodent models in which one-third of cortical COUP-TFII+ cells co-express SOX6 (Ma et al. 2012).

The subcortical septum is divided into MGE-like (NKX2.1 expressing) and LGE-like (PAX6 expressing) domains with OLIG2+ cells from this compartment migrating medially into the cortex (Alzu'bi et al. 2017). Here, we confirm expression of SP8 in LGE-like septum (Ma et al. 2013) and show that septal SP8+/CalR− cells also migrate toward the RMS in addition to dLGE derived SP8+ cells (Ma et al. 2013). However, COUP-TFI and COUP-TFII expression in both the MGE-like and LGE-like septum were confined to a very few, dispersed cells most likely to have migrated from other subcortical structures, making these septal compartments very similar to vMGE and dLGE, respectively.

The CGE is characterized by high expression of COUP-TFI and COUP-TFII, as well as SP8 and CalR. Dorsally, MGE-like CGE expresses NKX2.1, LGE-like CGE expresses PAX6 (Alzu'bi et al. 2017) but neither compartment expresses COUP-TFII in dividing cells of the VZ, which is only seen in the vCGE (Hansen et al. 2013; Ma et al. 2013; Alzu'bi et al. 2017). We have previously suggested that COUP-TFII+/CalR+ cells derived from the vCGE migrate into the cortex via the LGE (Alzu'bi et al. 2017). Here, we show that many of these cells also express COUP-TFI and SP8 and that numerous cells in the LGE co-expressing SP8 and COUP-TFII are passing through, rather than originating from the LGE as previously suggested (Ma et al. 2013).

### Migration Pathways out of the GEs

The idea that interneuron precursors principally enter the dorsal from the ventral telencephalon via 2 pathways; laterally from the MGE and caudally from the CGE (Faux et al. 2012; Marín 2013) has been challenged by recent observations. Medial migration of interneurons from the septum into the medial cortex has been described in shark (Quintana-Urzainqui et al. 2015) and human (Alzu'bi et al. 2017). Furthermore, studies in mouse (Touzot et al. 2016) and human (Alzu'bi et al. 2017) have suggested that CGE-derived interneurons reach their final location in the cortex via 2 distinct pathways. In addition to the caudal migratory stream (CMS) directing CGE-derived cells into the temporal cortex and hippocampus (Yozu et al. 2005) cells can also migrate anteriorly via the LGE into the anterior and lateral cortical regions. While COUP-TFII establishes the CMS (Kanatani et al. 2008) COUP-TFI is proposed to control the lateral/anterior migratory stream of CGE-derived cells in mice (Touzot et al. 2016).

The CGE is the source of neurons for various telencephalic structures (cortex, hippocampus, and amygdala) and multiple routes may be required for these cells to reach their specific targets (Touzot et al. 2016). Possibly, the anterior pathway for CGE-derived interneurons might be more important in human to facilitate these interneurons reaching the expanded and more evolved frontal cortex where the majority (50%) of calretinin-expressing interneurons reside (Ma et al. 2013; Hladnik et al. 2014). In addition, the temporal control of migration of later born CGE-derived interneurons allows proper laminar distribution in the cortex (Miyoshi et al. 2010; Touzot et al. 2016). We found that expression of COUP-TFI and COUP-TFII, and their downstream regulator SP8, are temporally distinct; however, the distribution of these 3 markers in each pathway may differ from what has been observed in mice (Touzot et al. 2016). The CMS seemed to be dominant at 8 PCW where cells mainly expressed both COUP-TFs but not SP8. However, SP8 was highly expressed in COUP-TFII+ cells in particular in the CMS at 12 PCW.

The anterior pathway for vCGE-derived cells via the LGE into the cortex became more prominent at older stages with COUP-TFII and SP8 more highly expressed than COUP-TFI in this migratory stream. In agreement with Ma et al. (2013) we observed that a proportion of SP8+ cells entering the cortex from the LGE appeared to originate locally in dLGE, however, these particular cells were negative for COUP-TFI, COUP-TFII, and CalR expression, suggesting that cells expressing any of these 3 markers with SP8 are uniquely generated in the vCGE. In addition, a proportion of COUP-TFI+ and COUP-TFII+ cells entering the cortex from the LGE could also have originated from the MGE/LGE boundary.

In rodents migrating interneurons disperse in the cortex via a superficial stream in the MZ and a deep stream in the lower IZ, although smaller numbers of cells also migrate through the SP (Lavdas et al. 1999; Marín and Rubenstein 2001; Marín 2013). The present study shows that the migration pattern of human cortical interneurons might be more dispersed with fewer distinguishable pathways than in rodents, at least at 8–12 PCW, where a significant number of GAD67+ cells also appeared to be migrating through the VZ and SVZ. In the SVZ and postmitotic layers, GAD67+ migrating cells co-expressed COUP-TFI, COUP-TFII, or SOX6. Interneurons in the VZ were solely MGE-derived cells co-expressing either SOX6 or COUP-TFI only (Fig. 6) whereas SP8 expression was confined to cells migrating in the SVZ/IZ but not in the MZ, SP, or VZ (Fig. 5; Ma et al. 2013). Therefore, the spatial origin and the expression of unique, or unique combinations of, transcription factors in migrating interneurons from the GE may control migration routes and thus their final laminar position in the developing cortex (Marín 2013) but this may be more complicated in human than in rodent models.

### Is Dorsal Interneurogenesis Limited to Subtypes Associated with the CGE?

To a large extent the organization and function of GABAergic cortical interneurons is shared between species, however, primates have a higher proportion of cortical interneurons (25–34% of cortical neurons) than rodents (15–25%) particularly in frontal cortex (reviewed by Hladnik et al. 2014) and there are functional differences between rodents and primates, including humans (Molnár et al. 2008; Povysheva et al. 2013; Clowry 2015). CalR+ GABAergic interneurons are more common in adult primates than in rodents (Conde et al. 1994; Gabbott et al. 1997; Barinka and Druga 2010). The ratio of parvalbumin-positive interneurons to projection neurons is similar in mouse and human but CalR positive interneurons have increased exponentially accounting for the overall increased proportion of interneurons in the human cortex (Hladnik et al. 2014). It is established that the human fetal brain has evolved an expanded and more complex outer SZV in the GEs (Hansen et al. 2013) and the CGE has increased in complexity extending ventrally as the temporal lobe has increased in size (Hansen et al. 2013; Ma et al. 2013; Alzu'bi et al. 2017). Together, these changes might underlie the relative increase in CGE-derived CalR+ interneurons. However, a more contentious proposal is that CalR+ interneurons, in particular, may be generated intracortically in primates especially at later developmental stages (Petanjek et al. 2009; Zecevic et al. 2011; Reinchisi et al. 2012; Hladnik et al. 2014; Radonjić, Ortega et al. 2014).

The present study shows that at 10–11 PCW a proportion of neurons derived from the cultured cortical progenitors are GABAergic and can co-express CalR, COUP-TFI, or COUP-TFII. We showed also that our cortical cultures retain positional information in terms of gene expression from the tissue from which they were cultured (also see Ip et al. 2010). This therefore provides another piece of evidence in favor of cortical interneurogenesis in human. There are 3 possible origins of GABAergic interneuron progenitor cells in culture; either (1) they are of cortical origin and/or (2) they are ventral progenitors that have migrated into the cortex retaining their proliferative capacity (Radonjić, Ayoub et al. 2014) or (3) as has been shown in previous rodent studies, cortical progenitors in vitro can “abnormally” generate GABAergic interneurons (Götz and Bolz 1994; He et al. 2001) possibly in response to exogenous factors (Trinh et al. 2006). For possibility (1) the lack of NKX2.1 expression in our cultures rules out cortex being able to produce interneurons associated with the MGE, such as basket cells and others, at this stage of development. However, for (2) COUP-TFI+ progenitors, that have downregulated NKX2.1, could have reached the cortex from dMGE and undergone further division. For (3) it is possible that FGF2 added to proliferating progenitors may have induced a ventral phenotype via induction of SHH signaling (Gabay et al. 2003). However, expression of FGF2 and its receptors is robust in developing human cortex in vivo (Ip et al. 2010; Lindsay et al. 2016) and cortical SHH expression increases with age (Miller et al. 2014; Radonjić et al. 2016) so that the conditions we employed in culture may not be so far removed from the in vivo condition. We propose that COUP-TFI and particularly COUP-TFII positive progenitors for GABAergic cells could have reached the cortex from the CGE, or be generated in the cortex and given the high levels of CalR expressed by GABAergic cells from cortical cultures it would seem that if cortical interneurogenesis exists it is primarily to contribute to CGE-like interneuron populations.

Finally, a higher proportion of COUP-TFII+ progenitors were present in anterior compared with posterior cortex derived cultures. This reflects previous qPCR studies suggesting elevated expression of various “GABAergic” genes in anteriorly including calretinin, *OLIG2*, *GAD,* and *DLX* genes (Ip et al. 2010; Al-Jaberi et al. 2015). The pathway from vCGE to anterior cortex via the LGE is no longer than that to the posterior cortex via the temporal cortex (Alzu'bi et al. 2017) and in the present study it was observed that the anterior pathway was increasing in numbers of migrating cells at the age at which these cultures were taken. Thus, an anterior preference for cortical interneurogenesis, at least over posterior if not temporal cortex, could be established by early migration of ventral progenitors to this region and/or by intracortical generation of GABAergic precursors.

## Conclusion

Both COUP-TFI and COUP-TFII are likely to play important roles in human forebrain development. In conjunction with other transcription factors, their expression helps delineate the protomap of the human cortex with COUP-TFI expression defining more posterior parts of the cortex but COUP-TFII confined to ventro-temporal cortex, which is relatively enlarged in human compared with other species. COUP-TFI expression defines nearly all the GE compartments contributing cortical interneurons whereas COUP-TFII is confined to the vCGE where distinct classes of interneurons more prominent in the primate brain are generated. Finally, COUP-TF expressing GABAergic neurons were generated from cortical progenitors in culture suggesting these transcription factors may direct the expansion of cortical interneuron populations in developing primate brain.

## Supplementary Material

Supplementary Data

Supplementary Data

Supplementary Data

Supplementary Data

Supplementary Data

Supplementary Data
