# Supplementary material for: The Transcription Factors COUP-TFI and COUP-TFII have Distinct Roles in Arealisation and GABAergic Interneuron Specification in the Early Human Fetal Telencephalon
Source: Cereb Cortex. 2017 Aug 9;27(10):4971–87. doi: 10.1093/cercor/bhx185 (PMC5903418; doi:10.1093/cercor/bhx185)
Supplement: Supplementary Data [file bhx185_supplementarymaterials.docx]

**Supplementary Materials**

| **Age (PCW)** | **Number of samples** | | | | |
| --- | --- | --- | --- | --- | --- |
|  | **Anterior** | **Central** | **Posterior** | **Temporal** | **Total** |
| **9-10** | 9 | 4 | 10 | 11 | 34 |
| **11-12** | 20 | 5 | 24 | 18 | 67 |
|  | | | | | **101** |

**Suppl. Table 1:** The number of samples of fetal cortex included at each age and location for the RNAseq study.

| **Primary antibody** | **Species** | **Dilution** | **Supplier** | **RRID number** |
| --- | --- | --- | --- | --- |
| KI67 | Mouse monoclonal | 1/150 | Dako, Ely, UK. | AB_2142378 |
| TBR1 | Rabbit polyclonal | 1/1000 | Abcam, Cambridge, UK. | AB_2200219 |
| TBR2 | Rabbit polyclonal | 1/200 | Abcam | AB_778267 |
| PAX6 | Rabbit polyclonal | 1/500 | Cambridge Bioscience, Cambridge, UK. | AB_2565003 |
| NKX2.1 | Mouse monoclonal | 1/150 | Dako | Not available |
| SOX6 | Rabbit polyclonal | 1/3000 | Abcam | AB_1143033 |
| COUP-TFI | Mouse monoclonal | 1/1500 | Abcam | AB_742210 |
| COUPT-FII | Mouse monoclonal | 1/500 | R&D Systems, Abingdon, UK. | AB_2155627 |
| OLIG2 | Rabbit polyclonal | 1/1000 | Merck Millipore, Watford, UK. | AB_10141047 |
| CalR | Mouse monoclonal | 1/2000 | Swant, Marly, Switzerland. | Not available |
| Calbindin | Rabbit polyclonal | 1/1000 | Swant | AB_10000340 |
| GAD67 | Mouse polyclonal | 1/1000 | Merck Millipore. | AB_2278725 |
| SP8 | Goat polyclonal | 1/500 | Santa Cruz, Heidelberg, Germany. | AB_2194626 |
| GABA | Rabbit polyclonal | 1/400 | Sigma-Aldrich, Poole, UK. | AB_477652 |
| GFAP | Rabbit polyclonal | 1/500 | Abcam | AB_305808 |
| β -tubulin III | Mouse monoclonal | 1/300 | Sigma-Aldrich | AB_477590 |
| β -tubulin III | Rabbit polyclonal | 1/300 | Abcam | AB_444319 |

**Suppl.Table 2**: Details of all primary antibodies used in the study.

**Suppl. Figure 1:** (A, A’) Double labelling for: COUP-TFI and the radial glia marker PAX6 in anterior (A) and posterior cortex (A’). (B, B’) COUP-TFI and intermediate progenitor marker TBR2 in anterior (B) and posterior cortex (B’). (C, C’) COUP-TFI and post-mitotic pyramidal neuron marker TBR1 in anterior (C) and posterior cortex (C’). The inset is drawing of 12 PCW sagittal sections with boxed areas where pictures (A-C’) were taken.

VZ: ventricular zone, SVZ: sub-ventricular zone, IZ: intermediate zone, pSP: pre sub-plate CP: cortical plate.

Scale bar = 200 μm

**Suppl. Figure 2:** The progenitor domains of COUP-TFI at 8 and 12PCW. (A-C) Double labelling for COUP-TFI and the cell division marker KI67 in the VZ/SVZ of the GE (A) anterior cortex (B) and posterior cortex (C) at 8 PCW. (D-F) Double labelling for COUP-TFI and KI67 in the LCGE (D) vCGE (E) but not dLGE (D) of 12 PCW. The insets are drawing of sagittal sections with boxed areas where images (A-C and D-E) were taken.

VZ: ventricular zone, SVZ: sub-ventricular zone, dCGE: dorsal CGE, vCGE: ventral CGE.

Scale bars = 500 μm in A; 100 μm in B (and for C); 200 μm in E (and for D, F).

**Suppl. Figure 3:** (A, B) Double labelling for COUP-TFI and NKX2.1 in the VZ/SVZ of the MGE of 8 PCW. Cells in the VZ are largely double-labelled, but the further into the SVZ, the more cells become labelled for either one transcription factor or the other (B). (C) shows double labelling for COUP-TFI and OLIG2 in the VZ/SVZ of the MGE of 8 PCW. In both cases double-labelling is confined to the dorsal MGE. The boxed area in (A) shows where image (B) was taken.

MGE: medial ganglionic eminence, LGE: Lateral ganglionic eminence

Scale bars = 500 μm in A and C; 20 μm in B.

**Suppl. Figure 4:** Double labelling for COUP-TFI (red) and COUP-TFII (green) in 8 and 12 PCW human fetal brain. (A) Sagittal section of 8 PCW fetal brain. The majority of cells in the caudal part of GE have shown co-localization of these two markers (yellow signal); while COUP-TFI was expressed in a decreasing posterior to anterior gradient; the anterior cortex was generally more populated with COUP-TFII+ cells than the posterior cortex however COUP-TFI (but not COUP-TFII) was markedly expressed in the anterior ventral pallium (B). Streams of COUP-TFI+ and COUP-TFII+ cells, and scattered COUP-TFI+/COUP-TFII+ cells appeared to migrate from the GE through the LGE toward the anterior cortex. (C, D) A proportion of cells in the anterior and posterior cortex also showed co-localization for these two markers. (E-J)Differing proportions of COUP-TFI+/COUP-TFII+ cells were observed in the CGE compartments (E, F) dLGE (G), ventral cortex (H), anterior cortex (I) and the posterior cortex (J) of 12 PCW fetal brain.

cp: choroid plexus, d Hip: dorsal hippocampus, dLGE: dorsal LGE, VZ: ventricular zone, SVZ: sub-ventricular zone, IZ: intermediate zone, CP: cortical plate, cp: choroid plexus, VP: ventral pallium, Pcrx: piriform cortex, dLGE: dorsal LGE, d and vCGE: dorsal and ventral CGE.

Scale bars = 500 μm in A; 200 μm in B; 100 μm in C (and for D); 200 μm in E (and for F, G); 200 μm in I (and for H, J).

**Suppl. Figure 5:** (A, B) SP8 was expressed in high dorso-rostral to low ventro-caudal gradient in cortical VZ, and in SVZ of the LGE and CGE at 8 PCW (A). Broad stream of SP8+ cells appearing to migrate ventrally from vCGE towards the posterior part of the mantle zone lateral and ventral to the GE, with only few SP8+ cells entering ventral-temporal cortex from the vCGE at this stage (B). (C-E) SP8 expression in coronal section of 12 PCW fetal brain (C), stream of SP+ cells appeared to be entering the cortex from dLGE (D), SP8 was also expressed in septum with stream of cells appearing to migrate ventrally and rostrally into the rostral migratory stream (RMS;E). (F, G) SP8 expression in sagittal section at 12 PCW (F), many SP8+ cells appeared to be entering the ventral-temporal cortex from vCGE at 12 PCW (G). Boxed area in A, C and F show where images (B, D, E and G) were taken.

ant: anterior, pos: posterior, Crx: cortex, Hip: hippocampus, Amy: amygdala, MGE: medial ganglionic eminence, dLGE: dorsal LGE, vCGE: ventral CGE, Sep: septum, RMS: rostral migratory stream, , LV: lateral ventricle.

Scale bars = 500 μm in A, C; 200 μm in B,E,G; 100 μm in D; 2mm in E.
