# Supplementary figures and images for: The Transcription Factors COUP-TFI and COUP-TFII have Distinct Roles in Arealisation and GABAergic Interneuron Specification in the Early Human Fetal Telencephalon
Source: Cereb Cortex. 2017 Aug 9;27(10):4971–87. doi: 10.1093/cercor/bhx185 (PMC5903418; doi:10.1093/cercor/bhx185)

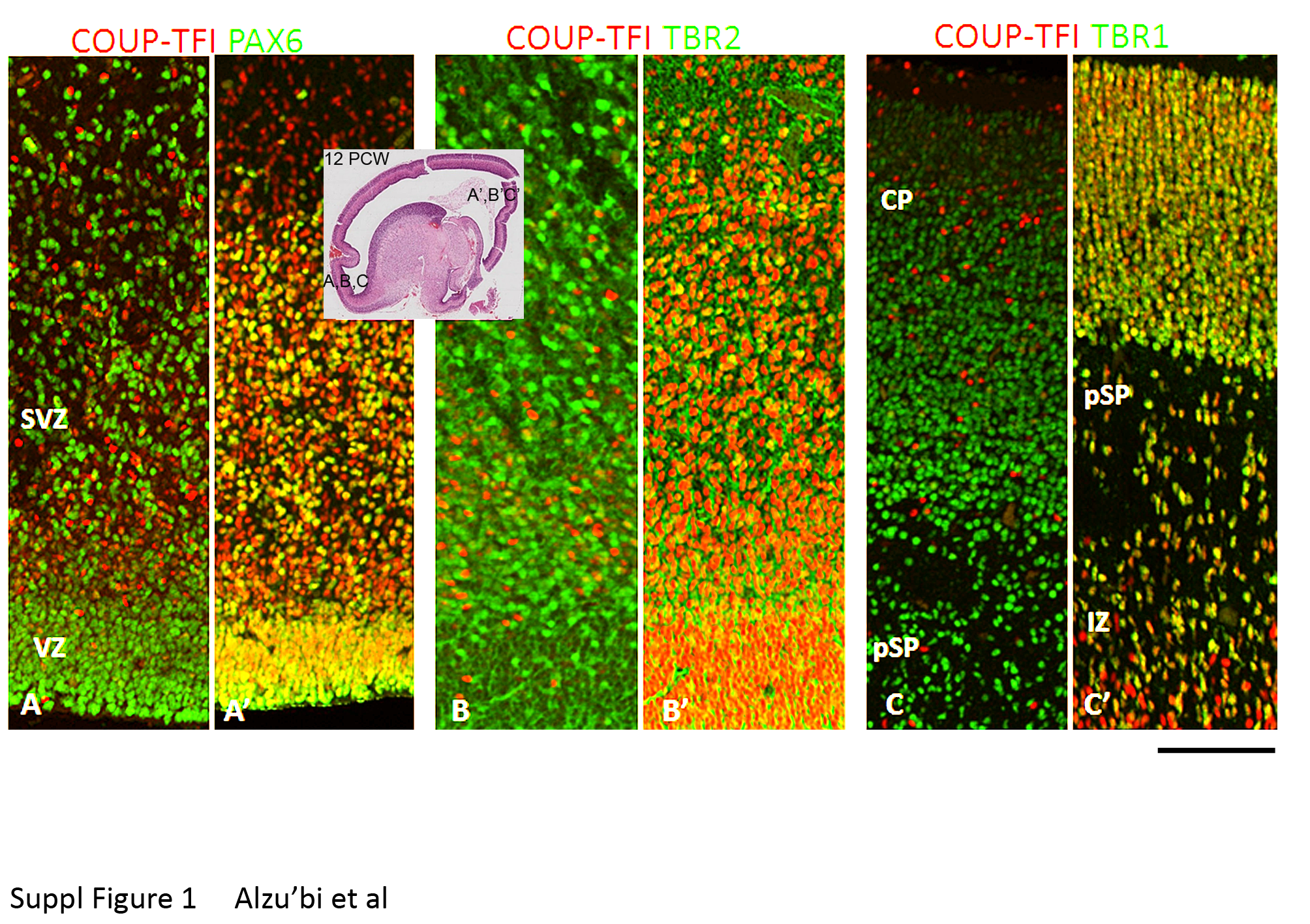

Supplement: Supplementary Data [file bhx185_supplfig1.png]

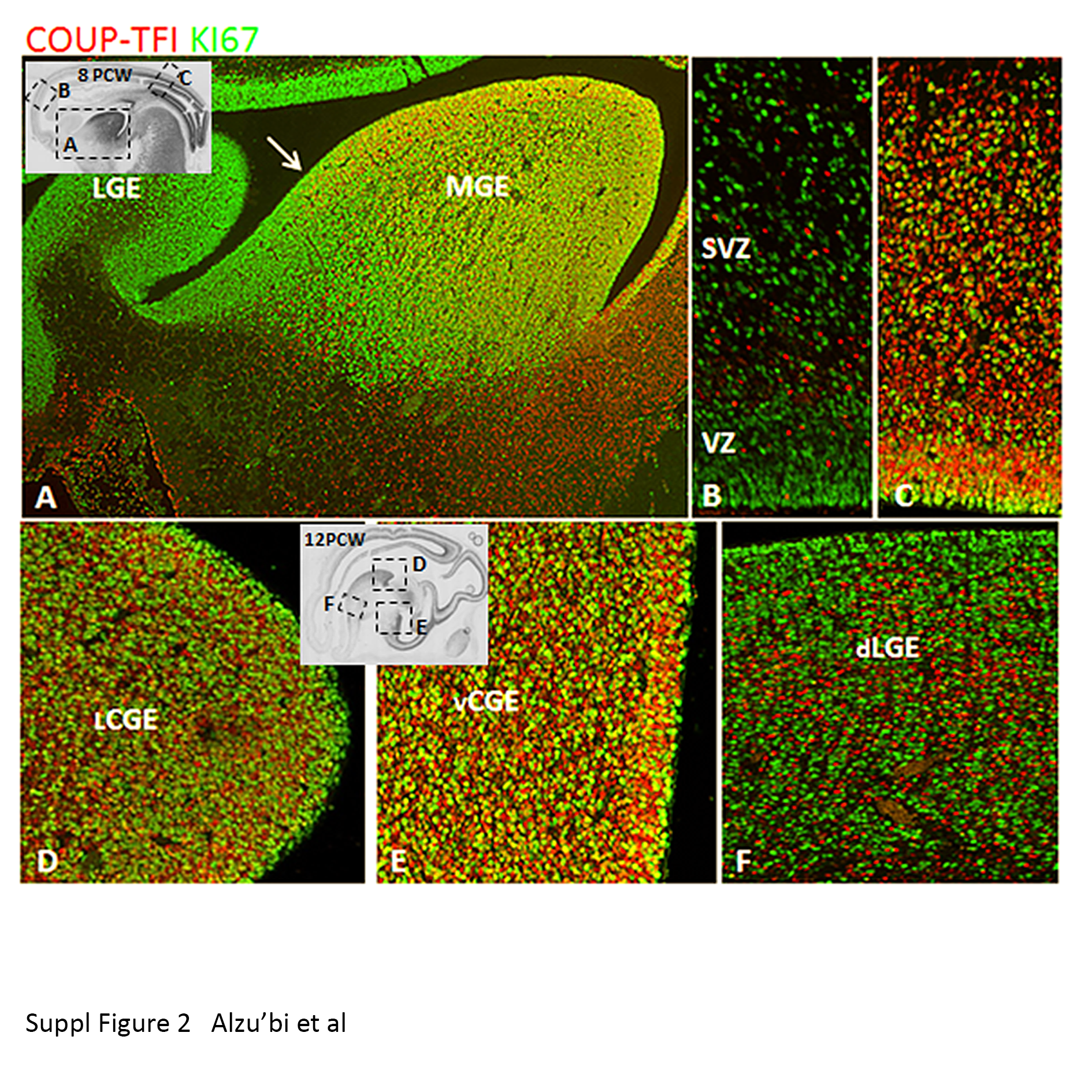

Supplement: Supplementary Data [file bhx185_supplfig2.png]

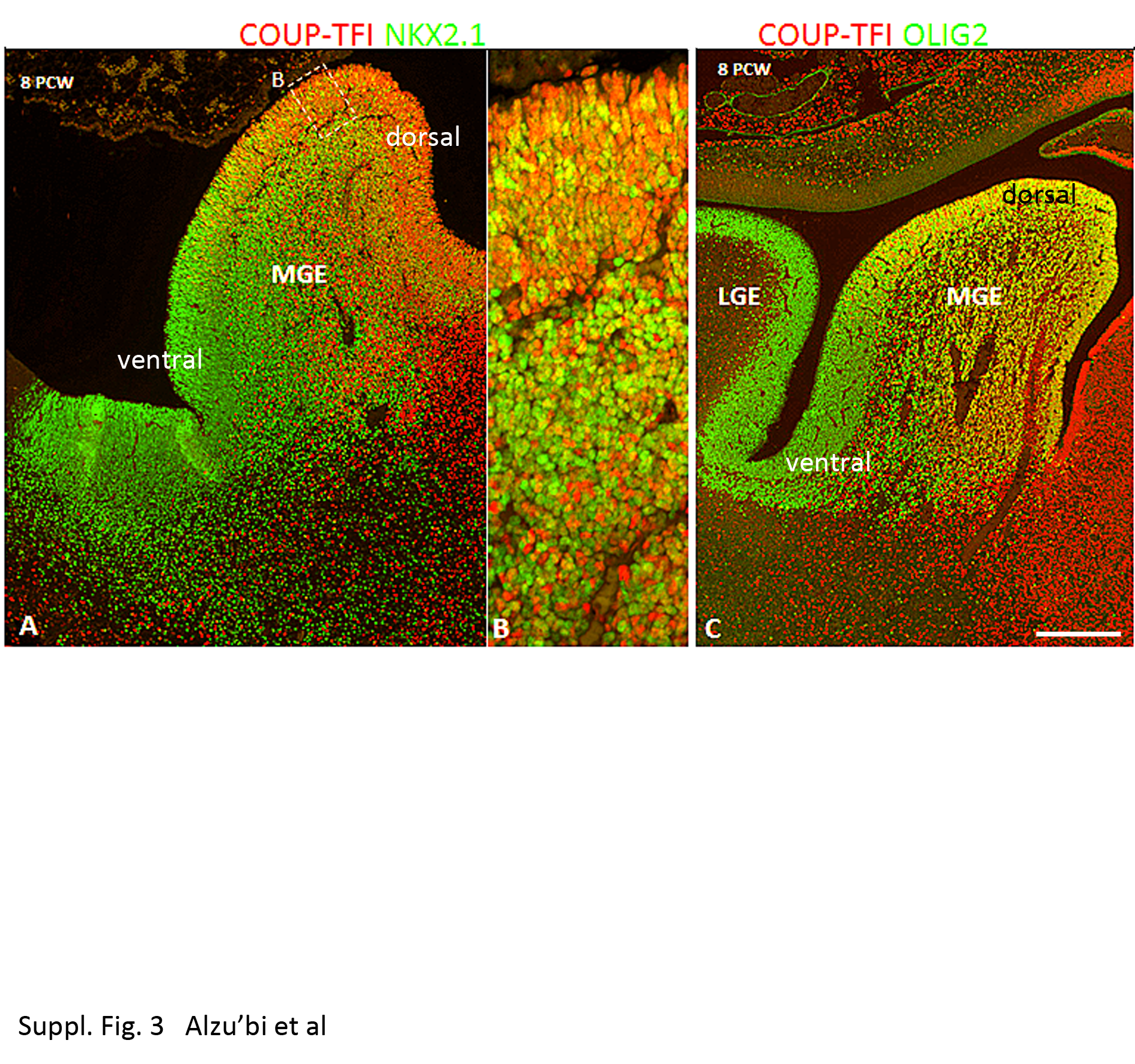

Supplement: Supplementary Data [file bhx185_supplfig3.png]

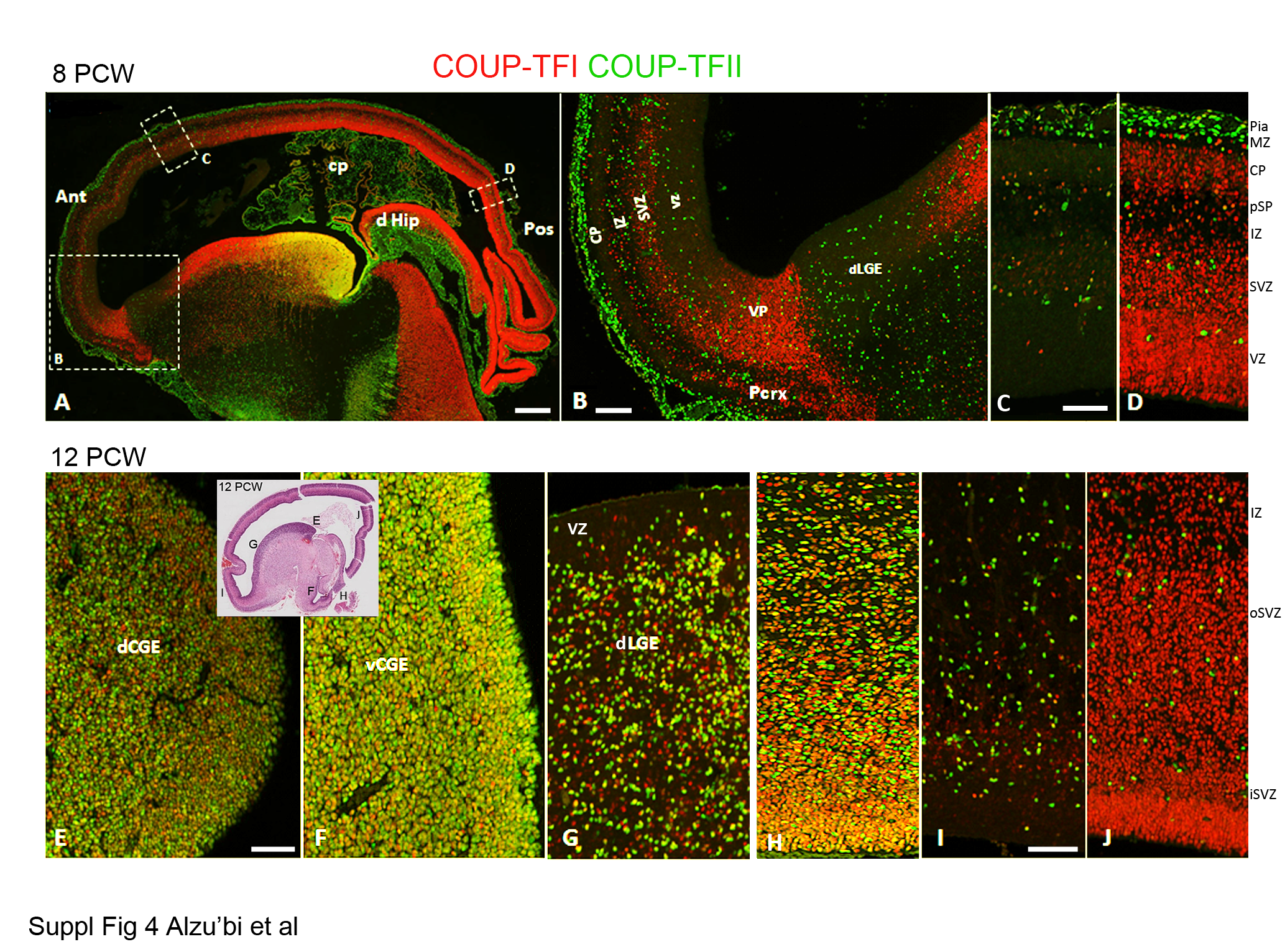

Supplement: Supplementary Data [file bhx185_supplfig4.png]

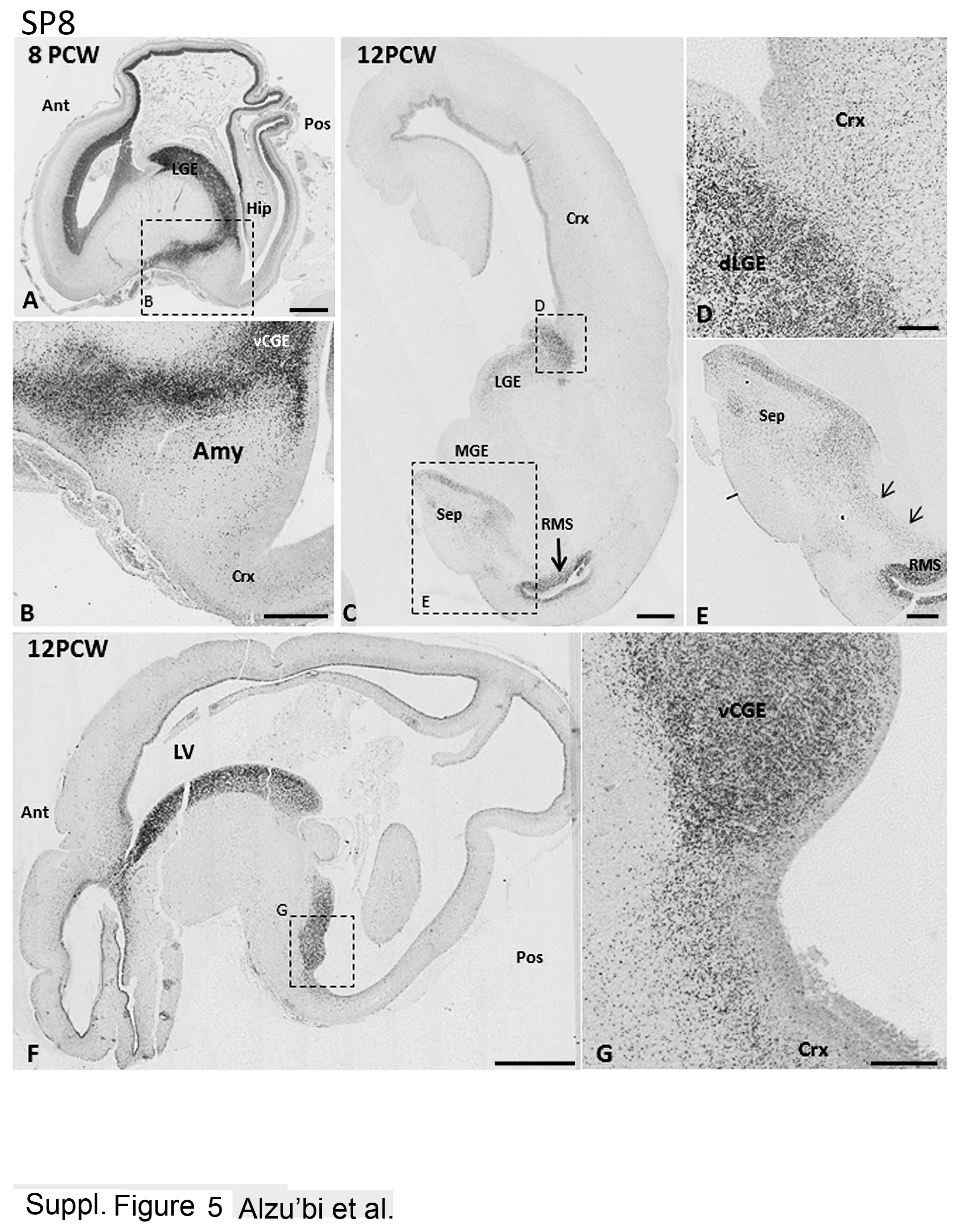

Supplement: Supplementary Data [file bhx185_supplfigure5.png]
